# All-trans retinoic acid induces reprogramming of canine dedifferentiated cells into neuron-like cells

**DOI:** 10.1371/journal.pone.0229892

**Published:** 2020-03-31

**Authors:** Rei Nakano, Taku Kitanaka, Shinichi Namba, Nanako Kitanaka, Masaki Sato, Yoshiyuki Shibukawa, Yoshikazu Masuhiro, Koichiro Kano, Taro Matsumoto, Hiroshi Sugiya

**Affiliations:** 1 Laboratory for Cellular Function Conversion Technology, RIKEN Center for Integrative Medical Sciences (IMS), Yokohama, Kanagawa, Japan; 2 Laboratory of Veterinary Biochemistry, College of Bioresource Sciences, Nihon University, Fujisawa, Kanagawa, Japan; 3 Department of Biology, Tokyo Dental College, Tokyo, Japan; 4 Department of Physiology, Tokyo Dental College, Tokyo, Japan; 5 Laboratory of Molecular and Cellular Physiology, College of Bioresource Sciences, Nihon University, Fujisawa, Kanagawa, Japan; 6 Laboratory of Cell and Tissue Biology, College of Bioresource Sciences, Nihon University, Fujisawa, Kanagawa, Japan; 7 Division of Cell Regeneration and Transplantation, Department of Functional Morphology, Nihon University School of Medicine, Tokyo, Japan; Laboratoire de Biologie du Développement de Villefranche-sur-Mer, FRANCE

## Abstract

The specification of cell identity depends on the exposure of cells to sequences of bioactive ligands. All-trans retinoic acid (ATRA) affects neuronal development in the early stage, and it is involved in neuronal lineage reprogramming. We previously established a fibroblast-like dedifferentiated fat cells (DFATs) derived from highly homogeneous mature adipocytes, which are more suitable for the study of cellular reprogramming. Canine cognitive dysfunction is similar to human cognitive dysfunction, suggesting that dogs could be a pathological and pharmacological model for human neuronal diseases. However, the effect of ATRA on neuronal reprogramming in dogs has remained unclear. Therefore, in this study, we investigated the effect of ATRA on the neuronal reprogramming of canine DFATs. ATRA induced the expression of neuronal marker mRNA/protein. The neuron-like cells showed Ca^2+^ influx with depolarization (50 mM KCl; 84.75 ± 4.05%) and Na^+^ channel activation (50 μM veratridine; 96.02 ± 2.02%). Optical imaging of presynaptic terminal activity and detection of neurotransmitter release showed that the neuron-like cells exhibited the GABAergic neuronal property. Genome-wide RNA-sequencing analysis shows that the transcriptome profile of canine DFATs is effectively reprogrammed towards that of cortical interneuron lineage. Collectively, ATRA can produce functional GABAergic cortical interneuron-like cells from canine DFATs, exhibiting neuronal function with > 80% efficiency. We further demonstrated the contribution of JNK3 to ATRA-induced neuronal reprogramming in canine DFATs. In conclusion, the neuron-like cells from canine DFATs could be a powerful tool for translational research in cell transplantation therapy, *in vitro* disease modeling, and drug screening for neuronal diseases.

## Introduction

The specification of cell identity during development depends on the exposure of cells to sequences of bioactive ligands (BLs). It has been reported that BLs mimic the developmental process and regulate the generation of specific neuronal subtypes from pluripotent stem cells (e.g., embryonic stem cells [ES cells] and induced-pluripotent stem cells). Furthermore, a previous study reported that several BLs are involved in neuronal development [1]. All-trans retinoic acid (ATRA) affects neuronal development in the early stage by controlling the generation of primary neurons [2–6]. In previous reports, ATRA induced neuronal lineage reprogramming in human ES cells, neural stem cells, and mouse embryonic fibroblasts. However, the effect of ATRA on neuronal specification and intracellular signaling has remained unclear [7–11].

The combination of cell-permeable small molecules has been reported to induce neuronal reprogramming. In mouse embryonic fibroblasts, four small molecules (forskolin, ISX-9, CHIR99021, and I-BET151; FICB) chemically induce neuronal cells [12], and in human adult fibroblasts seven small molecules (valproic acid, CHIR99021, repsox, forskolin, SP600125, GO6983, and Y-27632; VCRFSGY) are necessary for the induction of neuronal cells [13]. In human fetal and adult astrocytes, neuronal reprogramming was achieved by sequential exposure to nine (LDN193189, SB431542, TTNPB, thizovivin, CHIR99021, valproic acid, DAPT, SAG and purmorphamine) and six (valproic acid, CHIR99021, Repsox, Forskolin, ISX-9 and IBET-151) small molecules, respectively [14,15]. However, the underlying mechanisms and their reproducibility have remained obscure. In addition, it has been reported that the combination of these small molecules causes severe cell death [15]. Therefore, a simplified neuronal reprogramming model would be necessary for elucidating the mechanism of efficient reprogramming technology for functional neurons.

Previous studies have shown that adipose tissue can provide a cellular source for the study of cellular reprogramming. Adipose tissue contains nonadipocyte cells, known as the stromal-vascular fraction, which is isolated by centrifugation of collagenase-digested adipose tissue and comprised multipotent fibroblast-like cells known as adipose-derived stromal cells (ASCs). On the other hand, we established fibroblast-like cells derived from mature adipocytes and named the cells dedifferentiated fat cells (DFATs) [16–19]. DFATs showed the ability to differentiate into multiple mesenchymal cell lineages, indicating that DFATs are multipotent progenitor cells. DFATs have been obtained from a wide variety of mammals including mice, cats, dogs, and humans [16–20]. Although ASCs have been known to contain a variety of cell types, DFATs originate from a fraction of highly homogeneous mature adipocytes. The properties of DFATs (e.g. abundance, isolation method, robust proliferation capacity, and homogeneity) make them suitable for the study of cellular reprogramming [16–19].

The translation of rodent research into developing the treatment for human neuronal disease has been unsuccessful [21]. Neither neurobehavioral signs nor the development of natural cognitive dysfunction, (*e*.*g*. Alzheimer's disease (AD) and chronic stroke etc.) has been reported in the late life of rodents. The predicted outcomes in transgenic mouse models do not correlate with the findings of human clinical trials [22, 23]. On the other hand, canine cognitive dysfunction is a naturally occurring disease which is similar to human cognitive dysfunction. Like human’s, canine cognitive dysfunction is characterized by a progressive amnestic syndrome and AD-like pathology, and morbidity increase exponentially in old age [24, 25]. Therefore, dogs could be a pathological and pharmacological model for human neuronal diseases. Especially, cognitive dysfunction model using dogs would be a promising translational model to develop cellular transplantation therapies for unmet medical needs. However, it has been unclear if the simplified factor is effective for neuronal reprogramming in canine primary cultured cells [21]. Therefore, this study focused on canine DFATs and neuronal reprogramming.

In this study, ATRA activated the endogenous expression of neuronal marker genes, and induced functional conversion in canine DFATs into functional neuron-like cells. We further demonstrated the contribution of JNK3 to ATRA-induced neuronal reprogramming in canine DFATs.

## Materials and methods

### Materials

TRIzol, FITC-conjugated mouse monoclonal antibodies against CD29 (TS2/16), CD90 (5E10), CD14 (61D3) and HLA-DR (L243), FITC-conjugated rat monoclonal antibody against CD45 (YKIX716.13), PE-conjugated mouse monoclonal antibody against CD34 (1H6), PE-conjugated rat monoclonal antibody against CD44 (YKIX337.8), Alexa Fluor 594-conjugated F(ab′)2 fragments of goat anti-rabbit IgG (H+L), Alexa Fluor 488-conjugated goat polyclonal anti-rabbit IgG (H+L) cross-absorbed, ProLong Gold Antifade Reagent, Lipofectamine 2000, Neurobasal-A medium, B-27 supplement, and Opti-MEM were purchased from Thermo Fisher Scientific K.K. (Tokyo, Japan). CELLBANKER 1 plus medium, PrimeScript RT Master Mix, SYBR Premix Ex Taq II, Thermal Cycler Dice Real Time System II, and TP900 DiceRealTime v4.02B were obtained from TaKaRa Bio Inc. (Shiga, Japan). Anti-Neurofilament (2F11) mouse and anti-NeuroD2 (EPR5135), JNK1 (EPR140(2)), JNK2 (EP1595Y), and JNK3 (EPR5493(2)) rabbit monoclonal antibodies, veratridine, and FFN511 were purchased from Abcam (Cambridge, UK). Anti-nestin (Rat-401) mouse and anti-GFAP (D1F4Q) rabbit monoclonal antibodies were purchased from Cell Signaling Technology Japan, K.K. (Tokyo, Japan). Anti-β-actin mouse monoclonal antibody (AC74), ATRA, SP600125, MK2206, YM254890, JW67, FR180204, U0126, SB253080, SKF86002, AraC, atropine, d-tubocurarine, SCH23390, L-741626, and Triton X-100 were obtained from Sigma-Aldrich Inc. (St Louis, MO). Horseradish peroxidase-conjugated (HRP-conjugated) anti-rabbit and anti-mouse IgG antibodies, ECL Western Blotting Analysis System, and ImageQuant LAS 4000 mini were purchased from GE Healthcare (Piscataway, NJ). Anti-MAP2 mouse monoclonal (AP20) and anti-Ascl rabbit polyclonal antibodies were purchased from Merck KGaA (Darmstadt, Germany) and GeneTex, Inc. **(**Irvine, CA), respectively. Tetrodotoxin (TTX), 3,4-dihydroxyphenethylamine hydrochloride (dopamine hydrochloride), L-glutamiate, 4-aminobutyric acid (GABA), trypsin-EDTA, and Dulbecco's Modified Eagle’s Medium with 4.5 mg/mL glucose (DMEM-HG) were purchased from Wako Pure Chemical Industries Ltd. (Osaka, Japan). Acetylcholine chloride was purchased from Daiichi Sankyo Healthcare Co., Ltd. (Tokyo, Japan). iCycler, Mini-PROTEAN TGX gel, and polyvinylidene difluoride (PVDF) membranes were obtained from Bio-Rad (Hercules, CA). Complete mini EDTA-free protease inhibitor mixture, Block Ace, and collagenase II were purchased from Roche (Mannheim, Germany), DS pharma Biomedical (Osaka, Japan). Fluo-3-AM was purchased from Dojindo Lab. (Kumamoto, Japan). Fetal bovine serum (FBS) was purchased from Biowest (Nuaillé, France). A freezing vessel, BICELL, was purchased from Nihon Freezer Co., Ltd. (Tokyo, Japan). StatMate IV was purchased from ATMS (Tokyo, Japan).

### Primary dedifferentiated fat cell cultures

This study was approved by the Institutional Animal Care and Use Committee of HAMRI Co., Ltd. (16-H051). Three healthy beagle dogs (male, 1 year old, KITAYAMA LABES Co., Ltd.) were used in the experiments. This research was carried out in accordance with the Animal Research: Reporting of In Vivo Experiments guidelines. The dogs were bred and maintained in cages (height: 75 cm; width: 80 cm; and length: 75 cm). The experimental food (DS-A, 250 g/head, Oriental Yeast Co., Ltd.) was provided to all study animals once daily. The dogs were exercised using some toys inside (once a day) and outside (once a month) of the animal breeding facility. The physical conditions of the facility were monitored once daily. To avoid infection, the dogs were housed distantly from each other. All efforts were made to minimize animal discomfort.

DFATs were isolated by the ceiling culture method as previously described with slight modifications [16–20]. Briefly, canine celiac adipose tissue was minced in DMEM-HG containing 0.1% collagenase II and 2% FBS. The samples were incubated with gentle agitation for 30 min at 37°C and centrifuged at 500 × *g* for 3 min. The floating top layer containing mature adipocytes was isolated and collected. After three washes, the collected cells were transferred and ceiling cultured in a 25-cm^2^ culture flask with DMEM-HG supplemented with 20% FBS in an incubator at 37°C with 5% CO_2_. After three days, the cells were statically cultured under the same conditions before cryopreservation. The medium was changed twice weekly, and the DFATs were then obtained. The cells were harvested using 0.25% trypsin-EDTA once they reached approximately 90% confluence. The collected cells were seeded at a density of 1 × 10^6^ cells per 75-cm^2^ culture flask. The fourth-passage cells were used for all subsequent experiments.

### Primary cultured neurons and glial cells from canine cerebral cortex

Primary cultured neurons and glial cells were isolated by a previously described method with slight modifications [26, 27]. Cerebral tissues were collected and digested in dissociation media consisting of 0.25% trypsin in basal culture media at 37°C for 15 min. Following dissociation, the cells were plated at 2 × 10^6^ cells per 75-cm^2^ culture flask in Neurobasal-A medium containing 2% B-27 supplement for primary neurons. For the selection of primary neurons, 10 μM AraC was added after 48 h of incubation and the cells were cultured for 1 week in an incubator at 37°C with 5% CO_2_.

For primary glial cells, the cells were seeded at 2 × 10^6^ cells per 75-cm^2^ culture flask with DMEM-HG supplemented with 20% FBS in an incubator at 37°C with 5% CO_2_. The medium was changed twice weekly, and the cells were harvested using 0.25% trypsin-EDTA (Wako Pure Chemical Industries Ltd.) once they reached approximately 90% confluence. The collected cells were seeded at a density of 1 × 10^6^ cells per 75-cm^2^ culture flask. The fourth-passage cells were used for all subsequent experiments.

### Cell cryopreservation

The cells were harvested using 0.25% trypsin-EDTA once they reached 90–95% confluence. The collected cells were suspended in CELLBANKER 1 plus medium at a density of 2 × 10^6^ cells/500 μL, and 500 μL of the cell suspension was placed into each sterilized serum tube as previously described [28–35]. The tubes were then placed in a freezing vessel (BICELL) and cryopreserved at −80°C. Before the experiments, the serum tubes were removed from the BICELL vessel and immersed in a water bath at 37°C. The thawed cell suspension was transferred into a centrifuge tube containing growth medium and centrifuged at 300 × g for 3 min. After the removal of the supernatants, the pellet was suspended in growth medium and transferred to a 75-cm^2^ culture flask.

### ATRA-induced neuronal reprogramming

The cells were seeded at a density of 3 × 10^5^ cells/35-mm dish or 1 × 10^6^ cells/90-mm dish. Subsequently, the cells were treated with Neurobasal-A medium containing 2% B-27 supplement and 10 μM ATRA, and they were maintained in static culture in an incubator at 37°C with 5% CO_2_ for the indicated time periods. The medium was changed every 3 days.

### RT-qPCR

RT-qPCR was performed as previously described [28–35]. Total RNA was extracted from dermal fibroblasts with TRIzol reagent. First-strand cDNA synthesis was carried out with 500 ng of total RNA using PrimeScript RT Master Mix. RT-qPCR was performed with 2 μL of first-strand cDNA in 25 μL (total reaction volume) with SYBR Premix Ex Taq II and primers specific for *NF-H*, *Ascl1*, *NeuroD2*, *NF-L*, *NGFR*, *CHAT*, *TH*, *GLS*, *GAD*, and *TBP* (Table 1). RT-qPCR of no-template controls was performed with 2 μL of RNase- and DNA-free water. Additionally, RT-qPCR of no-reverse transcription control was performed with 2 μL of each RNA sample. The analysis was conducted using the Thermal Cycler Dice Real Time System II with the following protocol: 1 cycle of denaturation at 95°C for 30 s, 40 cycles of denaturation at 95°C for 5 s and annealing/extension at 60°C for 30 s. The results were analyzed by the crossing point method and the comparative cycle threshold (ΔΔCt) method using real-time RT-qPCR analysis software. The amplification of *TBP* from the same amount of cDNA was used as an endogenous control, and cDNA amplification from the cells at time 0 was used as a calibration standard.

**Table 1:** Primer sequences for RT-qPCR.

Gene	Gene bank ID	Primer sequences	Size (bps)
*NF-H*	NM_001003352.1	F: 5ʹ-GGAGGTTCCTGCCAAGGTGA-3ʹ	109
		R: 5ʹ-CTCTGCTGCTTTGCTGGGTTC-3ʹ	
*Ascl1*	XM_539745.3	F: 5ʹ-CAACTAAGCATTGCCTGCCTGA-3ʹ	154
		R: 5ʹ-CCCAGTGCCTTTGCACACA-3ʹ	
*NeuroD2*	XM_548146.3	F: 5ʹ-GGACTACAACAGCTCCGAGTACGA-3ʹ	133
		R: 5ʹ-CAGCGCCGAGTAGTGCATAGAA-3ʹ	
*NES*	XM_547531.2	F: 5ʹ-GGACGGGCTTGGTGTCAATAG-3ʹ	91
		R: 5ʹ-AGACTGCTGCAGCCCATTCA-3ʹ	
*GFAP*	XM_537614.2	F: 5ʹ-GCAGAAGTTCCAGGATGAAACCA-3ʹ	107
		R: 5ʹ-TCTCCAGATCCAGACGGGCTA-3ʹ	
*NF-L*	XM_534572.2	F: 5ʹ-TGAATATCATGGGCAGAAGTGGAA-3ʹ	140
		R: 5ʹ-GGTCAGGATTGCAGGCAACA-3ʹ	
*NGFR*	XM_548191.3	F: 5ʹ-ATGGGCAGCTCTCAGCCTGTA-3ʹ	105
		R: 5ʹ-AATGTAGGCCACTAAGCCCACAAC-3ʹ	
*CHAT*	XM_543902.3	F: 5ʹ-GACACACTGGTGGCCCAGAA-3ʹ	107
		R: 5ʹ-CGGCGGAAATTAATGACAACATC-3ʹ	
*TH*	NM_001002966.1	F: 5ʹ-AAGTTTGACCCTGACCTGGACTTG-3ʹ	100
		R: 5ʹ-GCTTGTATTGGAATGCGATCTCTG-3ʹ	
*GLS*	XM_545570.5	F: 5ʹ-GGGCACCACGACGTGTTTAAG-3ʹ	124
		R: 5ʹ-GCGGTTACAAGAGTCCGTCCA-3ʹ	
*GAD*	NM_001097543.1	F: 5ʹ-TGGAGCTGCACTTGGCTTTG-3ʹ	148
		R: 5ʹ-GCCAGCAGTTGCATTGACGTA-3ʹ	
*TBP*	XM_863452	F: 5'-ACTGTTGGTGGGTCAGCACAAG-3'	124
* *		R: 5'-ATGGTGTGTACGGGAGCCAAG-3'	

### Western blotting

Western blotting was performed as previously reported [28–35]. The cells were lyzed using lysis buffer containing 20 mM HEPES, 1 mM PMSF, 10 mM sodium fluoride, and a complete mini EDTA-free protease inhibitor cocktail at pH 7.4. Protein concentrations were adjusted using a previously reported method [37]. Extracted proteins were boiled at 95°C for 5 min in SDS buffer. Samples were loaded into separate lanes of a 7.5% or 12% Mini-PROTEAN TGX gel and separated electrophoretically. Separated proteins were transferred to PVDF membranes, treated with Block Ace for 50 min at room temperature, and incubated with primary antibodies (NF-H [1:500], Ascl1 [1:1,000], NeuroD2 [1:1,000], total JNK1 [1:1,000], total JNK2 [1:1,000], total JNK3 [1:1,000], and β-actin [1:10,000]) for 120 min at room temperature. After washing, the membranes were incubated with HRP-conjugated anti-rabbit or -mouse IgG antibody [1:10,000] for 90 min at room temperature. Immunoreactivity was detected using ECL Western Blotting Analysis System. Chemiluminescent signals were measured using an ImageQuant LAS 4000 mini (GE Healthcare).

### Flow cytometry

DFATs were characterized by flow cytometry analysis based on a previous report with slight modifications [26, 31]. Cells were placed in 5-mL round-bottom tubes at 3 × 10^5^ cells/tube with PBS containing 0.5% FBS, and incubated with antibodies (FITC-conjugated mouse monoclonal antibodies against human CD14, human CD29, human CD90, and human HLA-DR, FITC-conjugated rat monoclonal antibody against canine CD45, and PE-conjugated mouse monoclonal antibodies against canine CD34 and canine CD44) at 4°C for 30 min. An equal number of cells incubated with the respective isotype control antibodies were used as a control. Data were obtained by recording 10,000 events using EC800 (SONY, Tokyo, Japan), and analysis was performed with FLOWJO software (http://www.flowjo.com).

### Immunocytochemistry

Protein localization was investigated by immunocytochemical analysis as reported previously [26, 30, 36]. The cells were seeded at a density of 3 × 10^5^ cells/mL culture medium into a 35-mm glass bottom dish (Iwaki, Tokyo, Japan). The cells were fixed with 4% paraformaldehyde (Nacalai Tesque Inc., Kyoto, Japan) for 15 min. The fixed cells were permeabilized by incubation with 0.2% Triton X-100 for 15 min at room temperature. Non-specific antibody reactions were blocked for 30 min with Block Ace. The cells were then incubated for 90 min at room temperature with primary antibodies (NF-H, Ascl1, NeuroD2, and GABA [1:100]). After the cells were washed with PBS containing 0.2% polyoxyethylene (20) sorbitan monolaurate, they were incubated and visualized with Alexa Fluor 594-conjugated F(ab′)_2_ fragments of goat anti-rabbit IgG (H+L) [1:1,000] or Alexa Fluor 488-conjugated goat polyclonal anti-rabbit IgG (H+L) cross-absorbed [1:1,000] and TO-PRO-3-iodide [1:1,000] for 60 min in the dark at 25°C. The cells were also incubated with secondary antibodies alone as a control for non-specific binding. These samples were washed three times with PBS containing 0.2% polyoxyethylene (20) sorbitan monolaurate and visualized using a confocal laser scanning microscope (LSM-510; Carl Zeiss AG, Oberkochen, Germany).

### RNA-seq and data analysis

Total RNA was extracted from DFATs and ATRA-treated cells (28 days after treatment) using TRIzol reagent according to the manufacturer’s instructions. The quality of the RNA samples was checked using a nanodrop, agarose gel electrophoresis, and an Agilent bioanalyzer 2100 (Agilent Technologies, CA). The mRNA from eukaryotic organisms was enriched using oligo(dT) beads and fragmented randomly in fragmentation buffer, followed by cDNA synthesis using random hexamers and reverse transcriptase. After first-strand synthesis, a custom second-strand synthesis buffer (Illumina, NEB) was added with dNTPs, RNase H, and *Escherichia coli* polymerase I to generate the second strand by nick-translation. The final cDNA library was ready after a round of purification, terminal repair, A-tailing, ligation of sequencing adapters, size selection, and PCR enrichment. The library concentration was first quantified using a Qubit 2.0 fluorometer (Life Technologies), and it was then diluted to 1 ng/μl before checking the insert size on an Agilent 2100 followed by quantification by quantitative PCR (library activity > 2 nM) for greater accuracy. Libraries were fed into Illumina HiSeq 2000 machines (Illumina) according to activity and expected data volume. The original raw data from Illumina HiSeq 2000 were transformed to Sequenced Reads by base calling. Raw data were recorded in a FASTQ file, which contained sequence information (reads) and the corresponding sequencing quality information. Reference genome and gene model annotation files were downloaded directly from the genome website. The index of the reference genome was built using Bowtie v2.2.3 (http://bowtie-bio.sourceforge.net/bowtie2/index.shtml) and paired-end clean reads were aligned to the reference genome using TopHat v2.0.12 (https://ccb.jhu.edu/software/tophat/index.shtml). HTSeq v0.6.1 (https://htseq.readthedocs.io/en/release_0.10.0/) was used to count the read numbers mapped to each gene. The FPKM values were used to evaluate the expression levels of genes. Differential expression analysis was performed using the DESeq R package (1.18.0, https://bioconductor.riken.jp/packages/3.0/bioc/html/DESeq.html). DESeq provides statistical routines to determine the differential expression in digital gene expression data using a model based on the negative binomial distribution. The resulting *P*-values were adjusted using the Benjamini and Hochberg approach to control the false discovery rate. Genes with an adjusted *P*-value < 0.05 determined by DESeq were assigned as differentially expressed. The heat map, principal component analysis, hierarchical clustering, GO analysis, regional analysis, and pathway analysis were conducted using PANTHER 9.0 (http://www.pantherdb.org) and MeV 4.8.1 software (https://sourceforge.net/projects/mev-tm4/).

### siRNA transfection

Cells were transfected using Opti-MEM containing 5 μL/mL Lipofectamine 2000 and 50 nM JNK1, JNK2, JNK3, or scramble siRNA for 6 h [29–35]. The siRNA sequences are provided in Table 2. The siRNA efficiency was determined by western blotting.

**Table 2:** Sequences for siRNA transfection.

Gene	Gene bank ID	siRNA sequences
*JNK1*	XM_534943	GGACTTAAAGCCCAGTAAT
*JNK2*	XM_005626289	CTATTACCGGGCACCTGAA
*JNK3*	XM_005639148	GGAATAGTTTGCGCTGCGT

### Electrophysiological recordings

Conventional whole-cell voltage- and current-clamp recordings were performed as previously described [38]. All experiments were conducted at 25°C. Patch pipettes with resistances ranging from 3–7 MΩ were pulled from capillary tubes using a DMZ-Universal puller (Zeitz Instruments GmbH, Martinsried, Germany), and then back-filled with intracellular solution. Whole-cell currents were measured using a patch-clamp amplifier (L/M-EPC-7+; Heka Elektronik, Lambrecht, Germany) that allowed for compensation of the cell capacitance and series resistance. The currents (ramp currents) were elicited by voltage-ramp protocol from −80 mV to +60 mV (1.75 mV/ms) at a holding potential (Vh) of −70 mV. The currents were monitored and stored using pCLAMP software (Molecular Devices, LLC., CA) after digitizing the analog signals at 10 kHz (DigiData 1440A; Axon Instruments, Foster City, CA). The membrane capacitance of the cells was calculated based on capacitive transients evoked in 10-mV depolarizing steps from a holding potential (Vh) of 0 mV. Current amplitudes were normalized to these single-cell capacitance values and expressed as current densities (pA/pF). Action potentials were recorded by a patch clamp amplifier with a series of current step from 0 to 200 pA at a duration of 2,000-ms.

For patch-clamp recordings, the extracellular solution (ECS) consisted of the following: 137 mM NaCl, 5 mM KCl, 0.44 mM KH_2_PO_4_, 0.33 mM Na_2_HPO_4_, 10 mM glucose, 12 mM NaHCO_3,_ 0.5 mM MgCl_2_, and 10 mM HEPES, adjusted to pH 7.4 with tris(hydroxymethyl)aminomethane (Tris). To examine the Na^+^ selectivity of the currents, extracellular 136 mM NaCl was substituted with equimolar extracellular tetraethylammonium (Na^+^-free ECS). To record ionic currents under physiological conditions, intracellular solution containing 150 mM KCl, 10 mM HEPES, and 2 mM magnesium adenosine triphosphate adjusted to pH 7.2 with Tris was used. Recording solutions and the solution including 10 μM TTX were applied by superfusion over the cells using a rapid gravity-fed perfusion system (VC-6 or 8; Warner Instruments, Hamden, CT). Solution changes were completed within ~20 ms. The stock solution for TTX was prepared in distilled water and diluted with standard ECS to the appropriate concentration before use.

### Ca^2+^ imaging

The cells were seeded on 35-mm glass base dishes at a density of 4,000 cells/cm^2^. After 28 days of neuronal induction with or without ATRA, the cells were incubated with 4.0 μM Fluo-3-AM for 30 min at 37°C in the dark. Following incubation, the cells were washed twice with PBS. After washing, the culture medium was replaced with Ca^2+^ imaging buffer (containing 120 mM NaCl, 5 mM KCl, 0.96 mM NaH_2_PO_4_, 1 mM MgCl_2_, 11.1 mM glucose, 1 mM CaCl_2_, 1 mg/ml bovine serum albumin and 10 mM HEPES; pH 7.4). The glass base dishes with fluorescent dye-loaded cells were placed at room temperature on the stage of a confocal laser scanning microscope (LSM510). Frames in a time-lapse sequence were captured every 1 sec. After baseline images were acquired, the cells were stimulated with 50 mM KCl, 50 μM veratridine, 1 mM acetylcholine chloride, 100 μM dopamine hydrochloride, 1 mM GABA, or 100 μM L-glutamate. To examine the effects of antagonists, atropine (10 μM), d-tubocurarine (1 mM), SCH23390 (10 μM), and L-741626 (2 μM) were used as antagonists for the muscarinic, nicotinic, D1, and D2 receptors, respectively. After pretreatment for 5 min or 30 min with muscarinic and nicotinic or D1 and D2 antagonists, respectively, the cells were subsequently stimulated with 1 mM acetylcholine or 100 μM dopamine. The relative changes in intracellular Ca^2+^ concentrations over time were expressed as the relative change in baseline fluorescence.

### Fluorescent false neurotransmitter imaging

The cells were seeded on 35-mm glass base dishes at a density of 4,000 cells/cm^2^. After 28 days of neuronal induction with or without ATRA, the cells were incubated with 10 μM FFN511 for 30 min at 37°C in the dark. Following incubation, the cells were washed twice with PBS. After washing, the culture medium was replaced with imaging buffer (containing 120 mM NaCl, 5 mM KCl, 0.96 mM NaH_2_PO_4_, 1 mM MgCl_2_, 11.1 mM glucose, 1 mM CaCl_2_, 1 mg/ml bovine serum albumin, and 10 mM HEPES; pH 7.4). The glass base dishes with the fluorescent dye-loaded cells were placed at room temperature on the stage of a confocal laser scanning microscope (LSM510). Frames in a time-lapse sequence were captured every 1 s. After baseline images were acquired, the cells were stimulated with 50 mM KCl. The relative changes in neurotransmitter secretion over time were expressed as the relative change in baseline fluorescence. The fluorescence of the supernatant with or without 50 mM KCl treatment was measured using a fluorescence microplate reader (Fluoroskan Ascent FL, Thermo Fisher Scientific K.K.).

### Quantification and statistical analysis

The data from these experiments are presented as the mean ± standard error of the measurement. Statistical analysis was performed using StatMate IV (ATMS Co., Ltd., Tokyo, Japan). The data from the time course study were analyzed using two-way analysis of variance, and the data from other experiments were analyzed using one-way analysis of variance.

## Results

### Neuronal reprogramming of canine DFATs using ATRA

We isolated mature adipocytes from adult canine adipose tissue and established canine DFAT cells. Subsequently, we analyzed the expression of cell surface markers in DFATs by flow cytometry. DFATs were positive for CD29, CD44, and CD90, but negative for the hematopoietic lineage markers—CD14, CD45, and HLA-DR (S1 Fig). In this study, DFATs were distinctive from ASCs because of CD34 negative cells (S1 Fig), which is frequently used as a positive marker of adipose-derived mesenchymal stem cells. These results are consistent with previous reports [16–20, 39, 40], indicating that canine DFATs are homogenous and have distinctive properties from ASCs.

**Fig 1:**
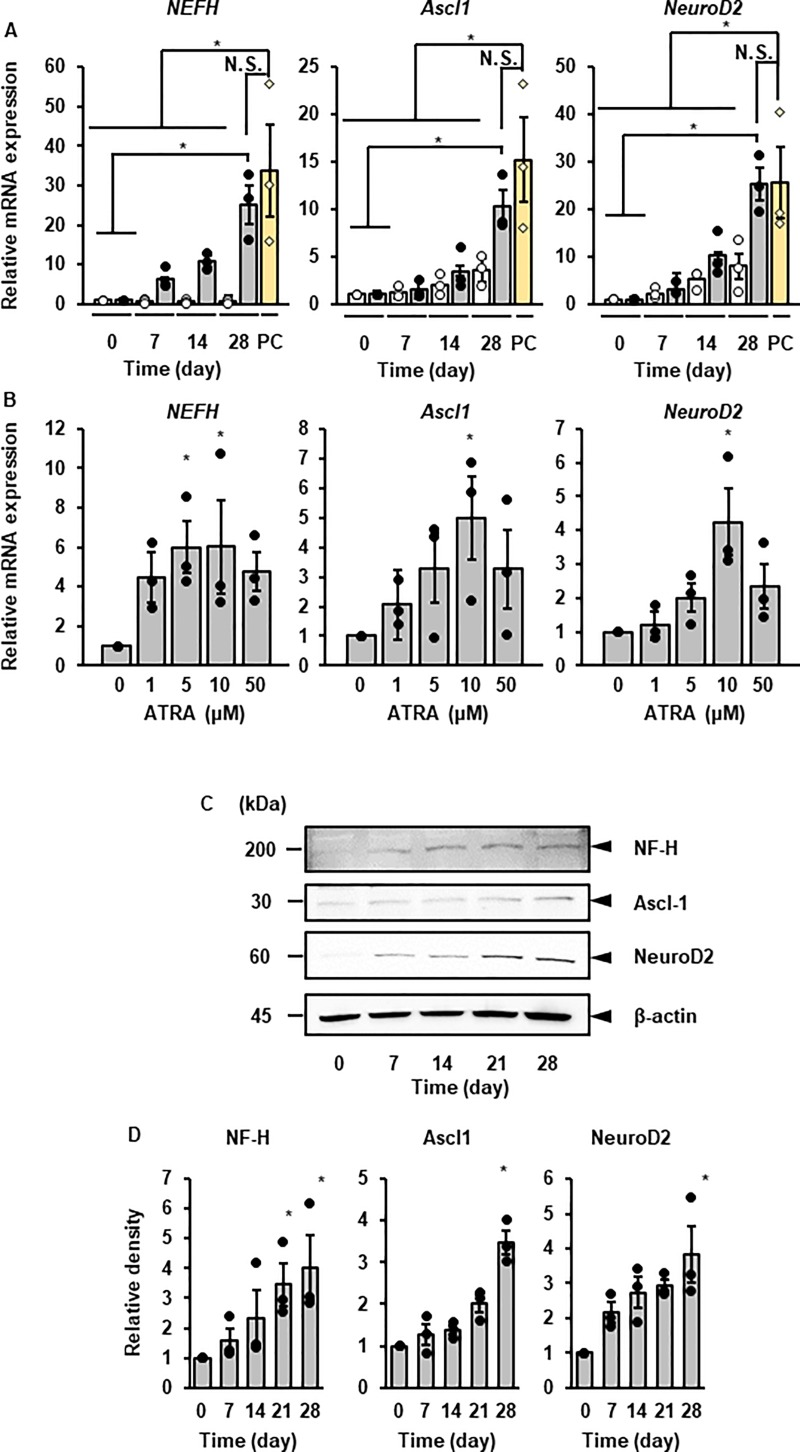
The effect of ATRA on the expression of neuronal marker mRNAs and proteins in canine DFATs. (A) Time-dependent changes in mRNA expression of the neuronal markers (NF-H, Ascl1, and NeuroD2) in ATRA-treated canine DFAT cells and positive control (PC; primary cultured neuron from canine cerebral cortex). DFATs were treated with (grey bar) and without (white bar) ATRA (10 μM). Data are shown as the mean ± standard error of three independent experiments. *P < 0.05. (B) The mRNA expression of NF-H, Ascl1, and NeuroD2 after 3 days of treatment with the indicated concentration of ATRA. Data are shown as mean ± standard error of three independent experiments. *P < 0.05, compared with 0 μM. (C) Representative images of protein expression of NF-H, Ascl1, and NeuroD2 after ATRA treatment. DFATs were treated with ATRA (10 μM). Cell lysate (10 μg protein) was applied to each lane. β-actin was used as an internal standard. (D) Relative density of the protein expression of NF-H, Ascl1, and NeuroD2 after ATRA. Data are shown as mean ± standard error of three independent experiments. *P < 0.05, compared with 0 day.

**Fig 2:**
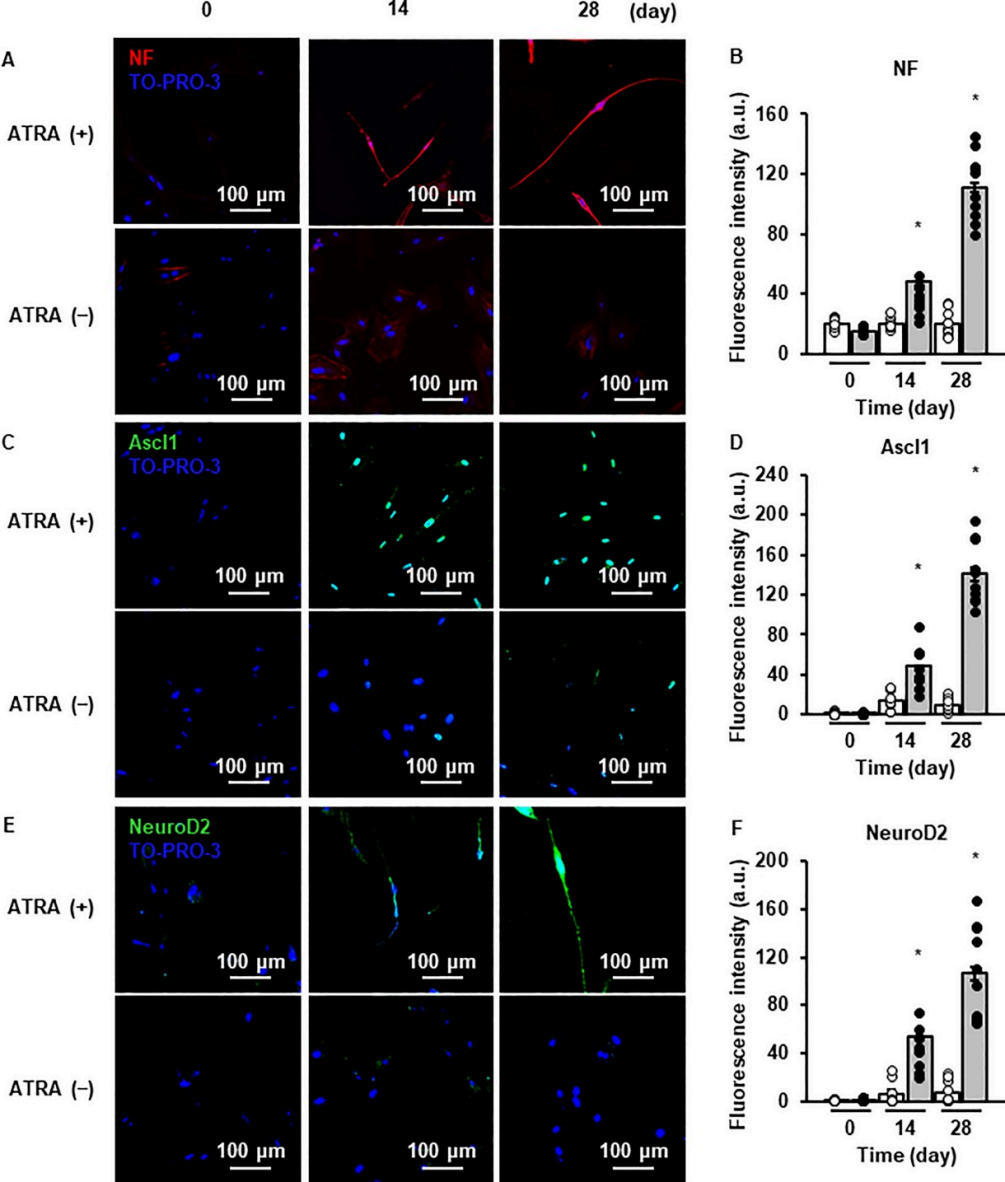
Immunocytochemical analysis of the localization of neuronal marker proteins. Representative images and fluorescence intensity of the cellular localization of NF-H (A and B), Ascl1 (C and D), and NeuroD2 (E and F) of DFATs (n = 10 cells, random selected ×20 fields from triplicate samples), with (upper panels) or without (control; lower panels) ATRA treatment after 0, 14, and 28 days. Data are shown as the mean ± standard error of three independent experiments. *P < 0.05, compared with 0 day.

ATRA induced the mRNA expression of the neuronal markers (*NF-H*, *Ascl1*, and *NeuroD2*) in canine DFATs in a time-dependent manner (Fig 1A). We compared the mRNA expression of *NF-H*, *Ascl1*, and *NeuroD2* in ATRA-treated cells with that in the primary cultured neurons from canine cerebral cortex (positive control; PC). There was no significant difference between the ATRA-treated cells and PC in terms of the mRNA expression of *NF-H*, *Ascl1*, and *NeuroD2* (Fig 1A). The cells treated with various concentration of ATRA (0–50 μM) for 3 days showed a dose-dependent induction of the mRNA expression of *NF-H*, *Ascl1*, and *NeuroD2* (Fig 1B). We found that 10 μM of ATRA was effective for the induction of the mRNA expression of *NF-H*, *Ascl1*, and *NeuroD2*, and this concentration was used for subsequent experiments. We observed that the protein expression of NF-H, Ascl1, and NeuroD significantly increased after ATRA treatment in a time-dependent manner by western blotting (Fig 1C and 1D). Immunocytochemical analysis showed that NF-H (Fig 2A and 2B) were localized in cell bodies and dendrites. The expression of Ascl1 (Fig 2C and 2D) and NeuroD2 (Fig 2E and 2F) was observed in the nuclei of ATRA-treated cells, whereas it was undetectable in untreated cells. We further checked the contamination of neural stem cells and glial cells in the ATRA-treated cells. The mRNA expression of the neural stem cell marker *NES* was stable in ATRA-treated cells and undetectable in primary cultured neuron from canine cerebral cortex (negative control; NC) (S2A Fig). The mRNA expression of glial marker *GFAP* was undetectable in ATRA-treated cells but expressed in primary cultured glial cells from canine cerebral cortex (PC) (S2B Fig). The protein expression of Nestin and GFAP was undetectable throughout the time course of the experiment (S2C Fig). These findings ruled out the contamination of neural stem cell and glial cell lineages in ATRA-treated cells. Consequently, it is more likely that canine DFATs are converted to neuron-like cells via an ATRA-induced neuronal reprogramming process.

### Functional properties of neuron-like cells from canine DFATs

**Fig 3:**
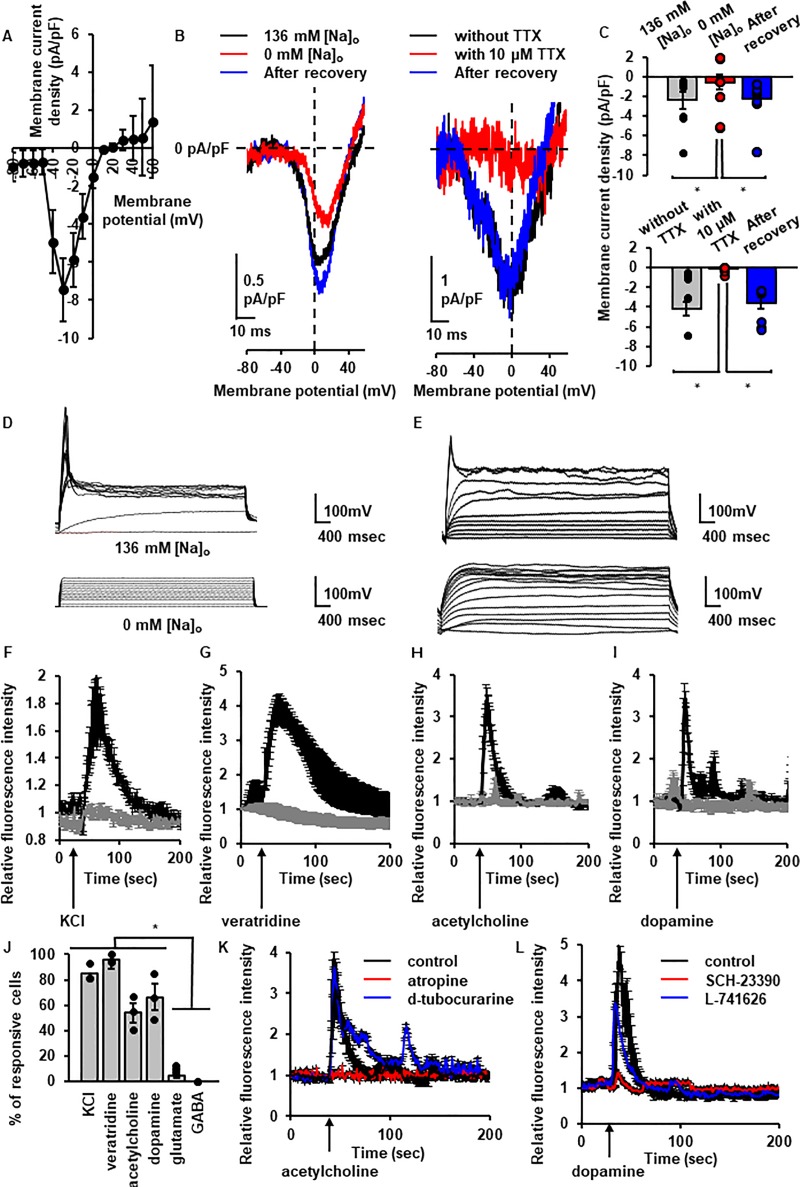
Electrophysiological properties and neurotransmitter response of ATRA-treated canine DFATs. (A) Average (mean ± standard error) current-voltage relationship (I-V curves) for Na^+^ currents of ATRA-treated canine DFATs (n = 3) in voltage-clamp mode. (B and C) The effect of removal of extracellular Na^+^ (B; left panel and C; upper panel) or tetrodotoxin (B; right panel and C; lower panel) on voltage-dependent Na^+^ currents. Representative traces (B) and averaged membrane current density (C) were recorded using the voltage-ramp protocol. Data are shown as mean ± standard error of three independent experiments. *P < 0.05. (D and E) The effect of removal of extracellular Na^+^ (D) or tetrodotoxin (E) on the generation of action potential. Representative traces in the presence (D; upper panel) or absence (D; lower panel) of extracellular Na^+^ were recorded using the current-clamp protocol. Representative traces with (E; lower panel) or without (E; upper panel) TTX (10 μM) were recorded using the current-clamp protocol. (F to I) Changes in the intracellular Ca^2+^ concentration evoked by KCl (F; 50 mM), veratridine (G; 50 μM), acetylcholine (H; 1 mM), and dopamine (I; 100 μM) in cells treated with (black line) or without (gray line) ATRA. (J) The percentage of cells that responded to the indicated stimulation (n = 30, random selected ×20 fields from triplicate samples). Data are shown as mean ± standard error of three independent experiments. *P < 0.05. (K) The effect of muscarinic receptor antagonist atropine (red line; 10 μM, 5 min) and nicotinic receptor antagonist d-tubocurarine (blue line; 1 mM, 5 min) on the changes in intracellular Ca^2+^ concentration evoked by acetylcholine (black line; 1 mM). Data are shown as mean ± standard error of three independent experiments. (L) The effect of pretreatment (30 min) with the D1 receptor antagonist SCH-23390 (red line; 10 μM) and the D2 receptor antagonist L-741626 (blue line; 2 μM) on the changes in intracellular Ca^2+^ concentration evoked by dopamine (black line; 100 μM). Data are shown as the mean ± standard error of three independent experiments.

To examine the electrophysiological properties of neuron-like cells from canine DFATs, we performed whole-cell patch clamp recordings. When the cells were voltage-clamped, fast inactivating inward currents were observed (Fig 3A). The inward current was completely and reversibly abolished in the absence of extracellular Na^+^ ([Na^+^]_o_) or in the presence of tetrodotoxin (TTX; 10 μM), suggesting that neuronal cells from DFATs exhibited TTX-sensitive voltage-dependent Na^+^ channels (Fig 3B and 3C). We further examined if the neuronal cells had an excitable membrane. In the current-clamp mode, we observed an action potential in neuronal cells after 21–28 days of ATRA treatment (Fig 3D and 3E, upper panel). The action potential was clearly attenuated in the absence of [Na^+^]_o_ or in the presence of TTX (Fig 3D and 3E, lower panel).

**Fig 4:**
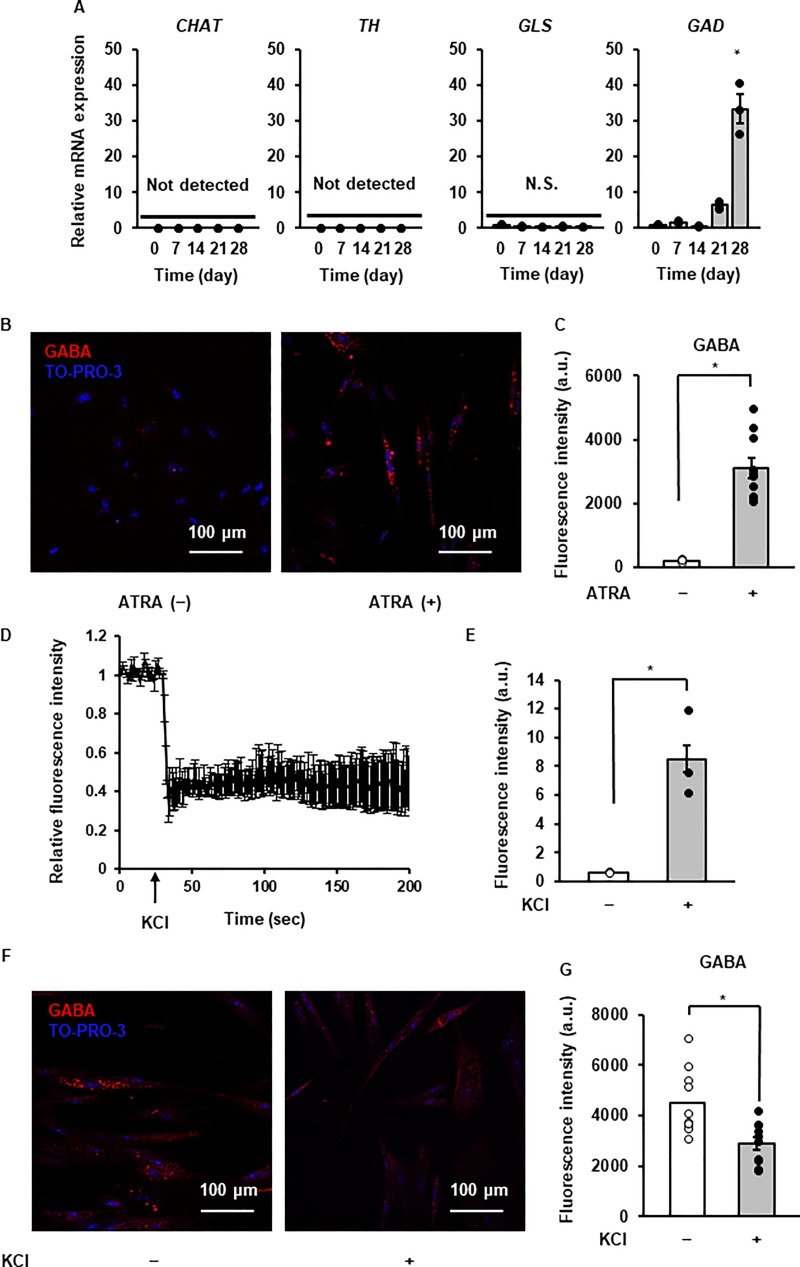
Neurotransmitter synthesis and secretion of ATRA-treated canine DFATs. (A) Time-dependent changes in the mRNA expression of enzymes for neurotransmitter synthesis after ATRA treatment. Data are shown as mean ± standard error of three independent experiments. *P < 0.05, compared with 0 day. (B) Representative images of GABA (red) and TO-PRO-3 (blue; nuclei) with (right panel) or without (left panel; control) ATRA treatment after 28 days. (C) Fluorescence intensity of GABA (n = 10 cells, randomly selected ×20 fields from triplicate samples) with or without ATRA treatment after 28 days. Data are shown as mean ± standard error of three independent experiments. *P < 0.05. (D) Destaining of fluorescent false neurotransmitter stimulated by KCl (50 mM) in ATRA-treated DFATs. (E) Fluorescence intensity of fluorescent false neurotransmitter in the culture supernatant of cells treated with or without KCl (50 mM). Data are shown as the mean ± standard error of three independent experiments. *P < 0.05. (F) Representative images of GABA destaining in ATRA-treated DFATs treated with (right) or without (left) KCl (50 mM). (F) Fluorescence intensity of GABA (n = 10 cells, randomly selected ×20 fields from triplicate samples) with or without KCl treatment after 28 days. Data are shown as the mean ± standard error of three independent experiments. *P < 0.05.

In a neuron, it is well known that an increase in intracellular Ca^2+^ concentration ([Ca^2+^]_i_) occurs via the activation of voltage-dependent Ca^2+^ channels (VDCC) when the cell is stimulated with an action potential and neurotransmitters. We observed that the effect of a high concentration of KCl (50 mM; 84.75 ± 4.05%), which activates VDCC, and the Na^+^ channel opener veratridine (50 μM; 96.02 ± 2.02%) induced an increase in [Ca^2+^]_i_ in Fluo3-loaded cells (Fig 3F and 3G). We further examined the effect of some neurotransmitters, acetylcholine, dopamine, L-glutamate, and γ-aminobutyric acid (GABA), on [Ca^2+^]_i_. The neuron-like cells were responsive to acetylcholine (66.26 ± 10.52%, Fig 4H) and dopamine (53.88 ± 7.71%, Fig 4I) but less to L-glutamate (9.72 ± 1.68%) and GABA (undetectable) (Fig 3J). Subsequently, to address the suitability of neuron-like cells from DFATs for drug discovery approaches, we investigated the subtypes of cholinergic and dopaminergic receptors using subtype-specific antagonists. As shown in Fig 3K and 3L, we identified that functional muscarinic and D1 receptors are dominantly expressed in neuronal cells from DFATs, highlighting that the cells were useful for cell-type-specific drug screening.

We further investigated neuronal subtypes based on the contents of neurotransmitters. ATRA dominantly induced mRNA expression of glutamic acid decarboxylase (*GAD*), a GABAergic neuronal marker (Fig 4A). Based on immunocytochemical analysis, GABA expression was observed in the ATRA-treated cell bodies, but not in the control (Fig 4B and 4C). Furthermore, we examined voltage-dependent neurotransmitter release in neuronal cells from DFATs using fluorescent analogs of neurotransmitters (fluorescent false neurotransmitter; FFN). VDCC was activated by a high concentration of KCl in FFN-loaded cells, and this leads to depolarization-induced FFN release (Fig 4D). FFN release in the culture supernatant was confirmed by measuring the increase in fluorescence intensity upon stimulation (Fig 4E). We also observed that the expression of GABA was reduced in response to KCl (Fig 4F and 4G). Taken together, we concluded that ATRA treatment produced acetylcholine- and dopamine-responsive GABAergic neuron-like cells from canine DFATs.

### Gene expression profiling of neuron-like cells derived from canine DFATs

**Fig 5:**
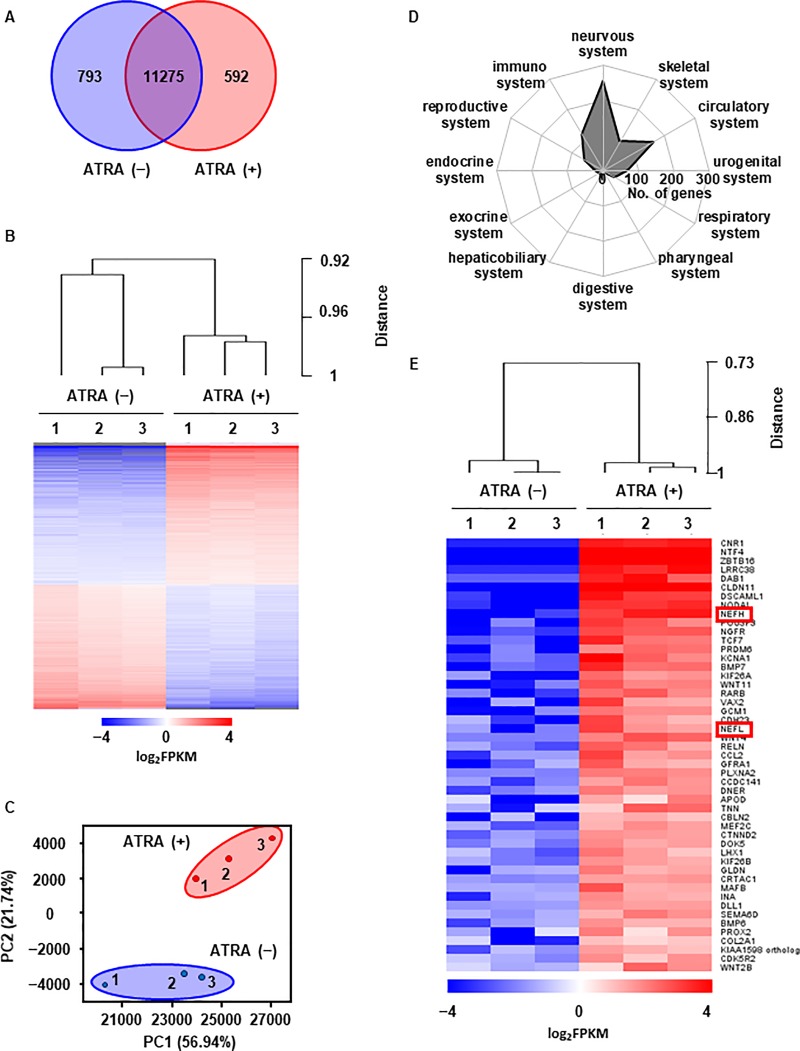
The effect of ATRA on gene expression profiling of DFATs. (A) Differentially expressed genes in canine DFATs treated with or without ATRA. (B) Hierarchical clustering and heat map of differentially expressed genes (*P* < 0.05) based on the RNA-seq data. The number above the heatmap indicates independent biological replicates. Red and blue indicate up-regulated and down-regulated genes, respectively. (C) Principle component analysis of samples from canine DFAT treated with or without ATRA (D) Radar chart showing the number of differentially expressed genes (*P* < 0.05) under GO terms related to the nervous system, skeletal system, circulatory system, urogenital system, respiratory system, pharyngeal system, digestive system, hepaticobiliary system, exocrine system, endocrine system, reproductive system and immune system in canine DFAT treated with or without ATRA. (E) Hierarchical clustering and heatmap illustrating the expression of the top 50 nervous system development genes in ATRA-treated DFATs. The number above the heatmap indicates independent biological replicates. Red and blue indicate up-regulated and down-regulated genes, respectively.

We compared the global gene expression patterns of DFATs (control) with that of ATRA-treated cells. The expression of 592 genes increased and that of 793 genes decreased in ATRA-treated cells compared with the control (Fig 5A). The global expression pattern with hierarchical cluster analysis and principal component analysis showed that ATRA-treated cells were distinct from the control, indicating that ATRA treatment induced drastic transcriptional changes (Fig 5B and 5C). Gene ontology (GO) analysis showed that the differentially expressed genes after ATRA treatment are involved in biological processes related to signaling, signal transduction, organ morphogenesis, cell communication, and developmental process (S3A Fig). To determine the reprogramming specificity toward the neuronal lineage, we investigated the expression of gene sets under GO terms reflecting the development of various tissue systems. We observed that ATRA treatment-induced transcriptional activation was predominantly directed towards the nervous system rather than other lineages (Fig 5D). In ATRA-treated cells, the expression of neuronal marker genes, including *NF-H* and *NF-L*, was up-regulated (Fig 5E) and validated by RT-qPCR (S3B Fig). However, the gene sets related to the GO terms of dedifferentiation, adipose tissue development, and glial cell development were almost unaltered (S3C Fig). These observations suggest that ATRA effectively induced neuron-like gene expression profile.

### Regional identity of neuron-like cells derived from canine DFATs

**Fig 6:**
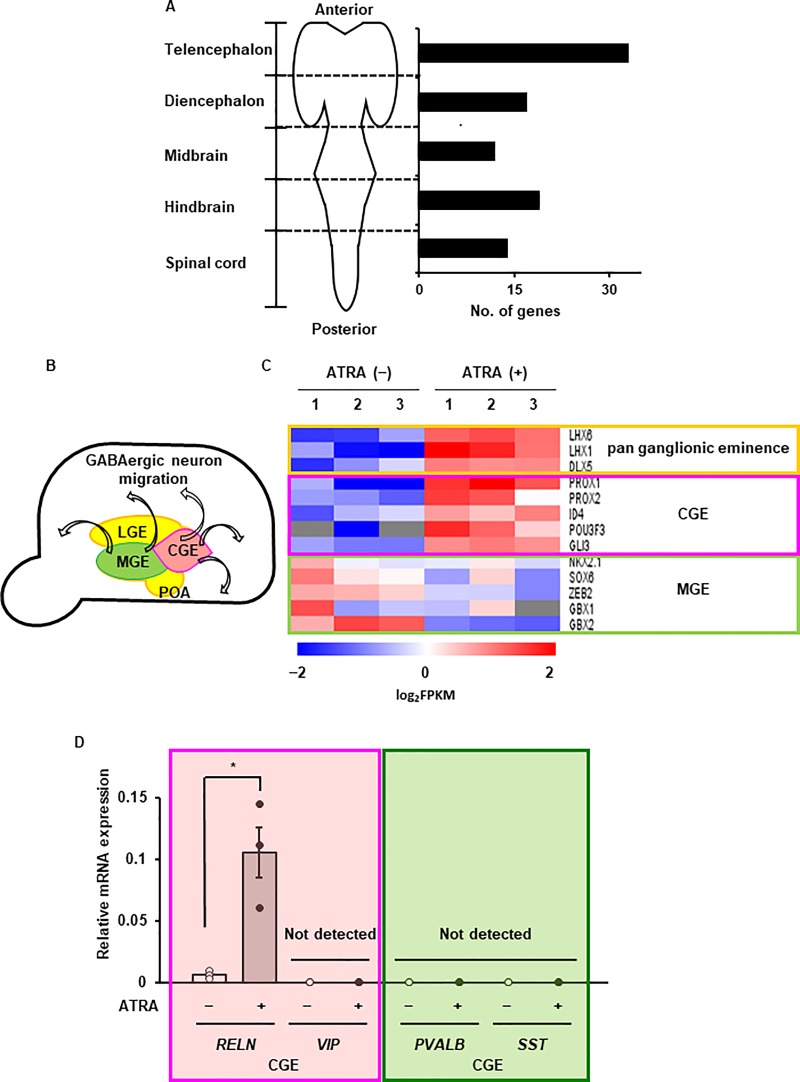
Regional identity of ATRA-treated canine DFATs. (A) The number of differentially expressed regional genes (*P* < 0.05) in ATRA-treated DFATs. (B) Schematic diagram illustrating that the medial ganglionic eminence (MGE) and the caudal ganglionic eminence (CGE), but not the lateral ganglionic eminence (LGE) and preoptic area (POA), are the primary sources of cortical GABA interneurons. (C) Heatmap illustrating the expression of the pan ganglionic eminence, MGE and CGE genes, in canine DFATs with or without ATRA treatment. (D) The expression of the cortical interneuron subtype markers reelin (RELN), vasoactive intestinal peptide (VIP), parvalbumin (PVALB), and somatostatin (SST). Data are shown as the mean ± standard error of three independent experiments. *P < 0.05.

To determine the regional identity of neuron-like cells from canine DFATs, we investigated the expression of regional markers along the anterior-posterior axes of neuronal development. We observed that the expression of telencephalon marker genes, such as *POU3F3*, *RARB*, and *RELN*, significantly increased, whereas the expression of other regional markers was less altered (Fig 6A). A wide variety of GABAergic interneuron subtypes were localized in the neocortex. Cortical interneurons develop in the ventral telencephalon and transmigrate into the cortex. As summarized in Fig 6B., the ventral telencephalon is characterized by three morphologically distinct regions: lateral, medial, and caudal ganglionic eminences (LGE, MGE, and CGE), and preoptic area (POA). In a previous study, *in vivo* fate mapping showed that MGE and CGE, but not LGE and POA, are the primary sources of cortical interneurons. MGE produces ~70% of the total GABAergic cortical interneuron population, specifically parvalbumin (*PVALB*)- or somatostatin (*SST*)-expressing cells, whereas interneurons from CGE comprise ~30% of the total interneurons, the majority of which express reelin (*RELN*) or vasoactive intestinal polypeptide (*VIP*). The distinct TFs control the subtype specification of MGE- or CGE-derived cortical interneurons. Our results showed that the expression of the master transcriptional factors for CGE, such as *PROX1/2*, *ID4*, *POU3F3*, and *GLI3*, significantly increased. In addition, we observed that the expression of RELN was dominantly increased after ATRA treatment (Fig 6C and 6D). These results suggest that ATRA induces the reprogramming of canine DFATs into *RELN*-expressing and CGE-derived GABAergic cortical interneuron-like cells. Taken together, ATRA evoked the neuron-subtype specific intrinsic reprogramming process in canine DFATs and subsequent reprogramming into GABAergic cortical interneuron-like cells.

### Contribution of JNK subtype to ATRA-induced neuronal reprogramming

**Fig 7:**
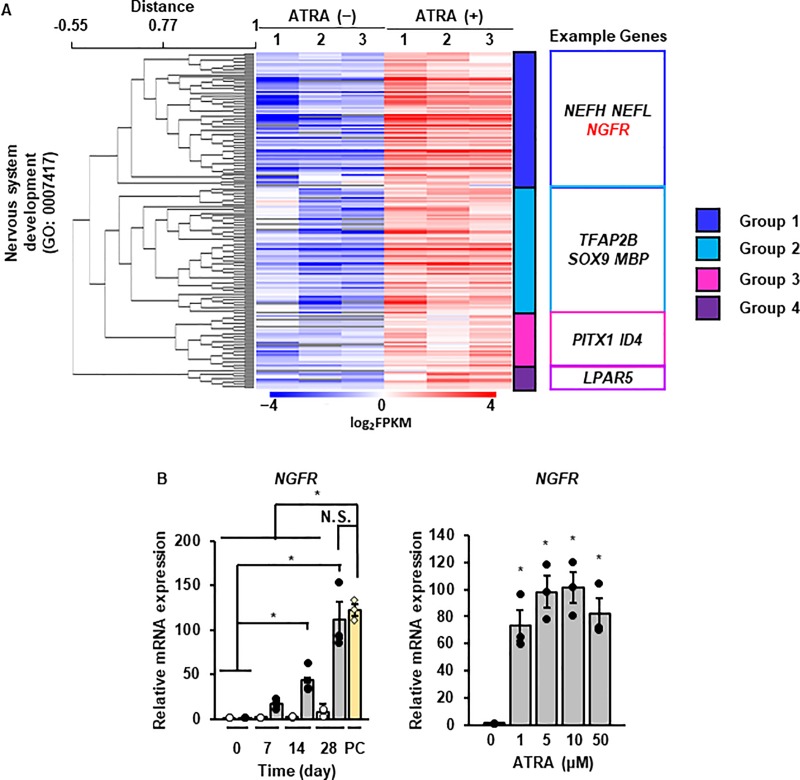
*NGFR* as a marker for ATRA-induced neuronal reprogramming. (A) Heat map showing unsupervised hierarchical cluster analysis of up-regulated genes (*P* < 0.05) under GO terms of nervous system development. The number above the heat map indicates independent biological replicates. Example genes (right) of each group are shown. Red and blue indicate up-regulated and down-regulated genes, respectively. (B and C) Time (B) and dose (C)-dependent changes in *NGFR* mRNA in DFATs treated with ATRA at the indicated time points and the indicated concentration of ATRA. Data are shown as the mean ± standard error of three independent experiments. *P < 0.05, compared with 0 day (B) or 0 μM (C).

To monitor the neuronal reprogramming process, we further analyzed the up-regulated gene sets related to nervous system development. The up-regulated genes could be classified into four groups by unsupervised hierarchical cluster analysis, and the typical neuronal marker genes were classified into group 1 (Fig 7A and S4 Fig). We confirmed that the expression of *NGFR* mRNA was induced in neuronal cells from DFATs (Fig 7B). These results support the notion that NGFR is a sensitive marker for neuronal reprogramming process in this system. To clarify the intracellular signaling pathways that contribute to ATRA-induced neuronal reprogramming, we used *NGFR* as a marker for subsequent experiments.

**Fig 8:**
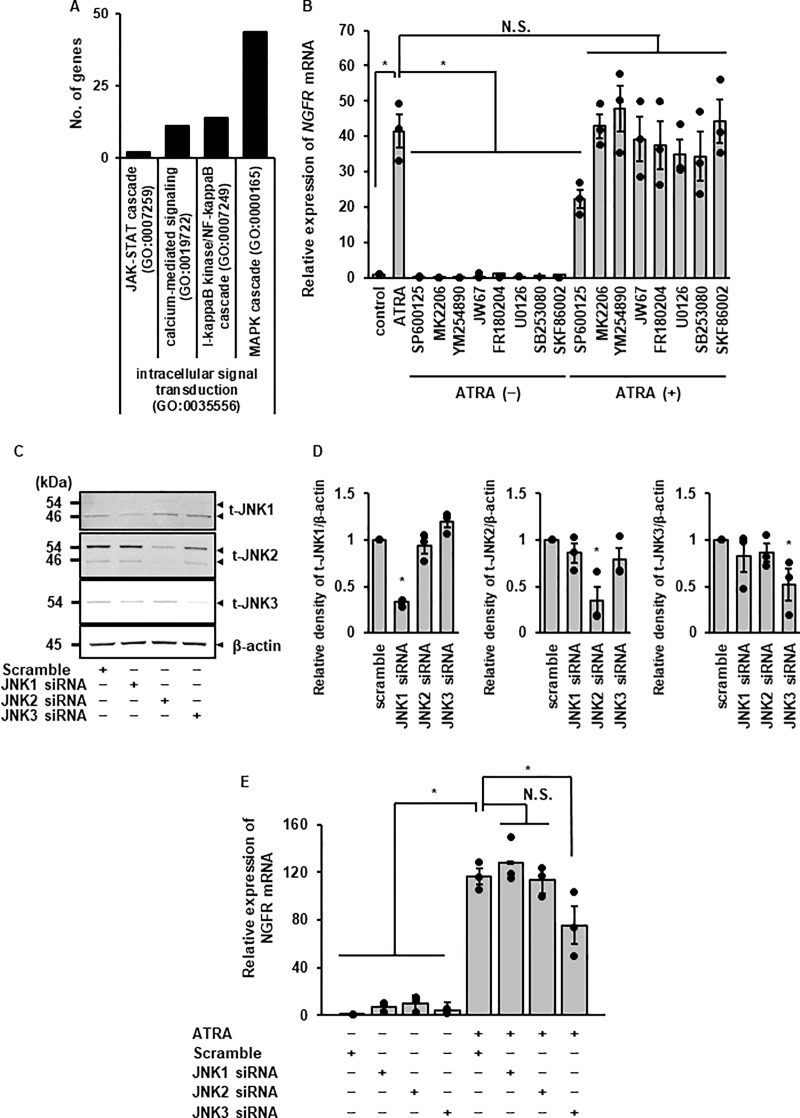
Contribution of JNK subtype to ATRA-induced neuronal reprogramming. (A) Pathway analysis showing the number of differentially expressed genes (*P* < 0.05) under GO terms of intracellular signal transduction. (B) The effect of inhibitors on ATRA-induced expression of *NGFR*. Data are shown as the mean ± standard error of three independent experiments. *P < 0.05. (C to D) Protein expression of JNK1, 2, and 3 in JNK1, 2, or 3 siRNA-transfected cells. JNK1, 2 or 3 siRNA transfection resulted in a significant decrease in the expression of JNK1, 2, or 3 protein, respectively, but not scramble siRNA transfection (C). The relative density of JNK1, 2, or 3 protein expression in siRNA-transfected cells compared with scramble siRNA-transfected cells is illustrated (D). β-actin was used as an internal standard. Data are shown as the mean ± standard error of 3 independent experiments. *P < 0.05. (E) Decrease in ATRA-induced *NGFR* mRNA expression in cells transfected with JNK3 siRNA, but not in those transfected with JNK1 or 2 siRNA. Data are shown as the mean ± standard error of three independent experiments. *P < 0.05.

Furthermore, we performed pathway analysis using RNA-seq data. The pathway analysis showed that MAPK cascade was dominantly activated in ATRA-treated cells (Fig 8A). Furthermore, we screened the effect of several inhibitors on ATRA-induced *NGFR* mRNA expression. ATRA-induced *NGFR* mRNA expression was attenuated in cells pre-treated with the JNK inhibitor, SP600125, whereas the inhibitors for other pathways, including other MAPKs (p38 and ERK) were less effective (Fig 8B). To confirm the crucial role of JNK in neuronal reprogramming, we performed JNK knockdown experiments using siRNA transfection. Currently, three mammalian JNK genes, *JNK1*, *JNK2*, and *JNK3*, are known to specify JNK isoforms **[52–54]**. In DFATs, all the JNK subtypes were detected, and the expression of each subtype was decreased in the cells transfected with JNK1, 2, and 3 siRNA, respectively, whereas transfection with scramble siRNA as a control had no effect (Fig 8C and 8D). In such knockdown experiments, transfection with JNK3 siRNA resulted in the attenuation of ATRA-induced *NGFR* mRNA expression, but not with JNK1, JNK2, and scramble siRNAs (Fig 8E). Taken together, these results show that JNK3 may be involved in ATRA-induced neuronal reprogramming process.

## Discussion

ATRA has been reported to be involved in neuronal development, especially in GABAergic specification [41, 42]. In this study, the addition of ATRA to the culture medium throughout the neuronal reprogramming processes was required for the induction of neuronal marker expression and neuronal function in canine DFATs. This was consistent with a previous report that ATRA induces neuronal lineage reprogramming in several types of cells [7–11]. However, in human fetal and adult astrocytes, ATRA is dispensable for neuronal reprogramming, and the mechanisms responsible for astrocyte-to-neuron reprogramming are thought to differ from those of neuronal development, suggesting that the context and types of starting cells are partly involved in the efficacy of neuronal reprogramming strategy [14, 15]. Taken together, these suggest that ATRA plays a crucial role in the neuronal reprogramming process in canine DFATs.

The generation of specific neuronal cell types has remained challenging. Cortical GABAergic interneurons are the key neuronal subtypes for a balanced neural excitation within the brain [43]. The dysfunction of GABAergic interneurons is directly and indirectly involved in many neuronal diseases, such as Huntington’s diseases, autism, schizophrenia, bipolar depression, and epilepsy [44]. The generation of high-purity GABAergic interneurons is necessary for studies on the pathophysiology of incurable neuronal diseases and in drug discovery research. In previous studies, GABAergic neurons were differentiated from pluripotent stem cells (e.g., ES cells and iPS cells) [45–48]. It has also been reported that fibroblasts and pluripotent stem cells are directly converted into GABAergic neurons by ectopic expression of a set of cell-fate-determining transcriptional factors. However, most of these methods have multiple intermediate stages and result in contamination by non-neuronal cells. The protracted timeline required to attain neuronal function is a further limitation because the process takes as long as 8 weeks [48]. In this study, we demonstrated that GABAergic cortical interneuron-like cells can be efficiently reprogrammed from canine DFATs by treatment with ATRA within 4 weeks. Our results showed that > 80% of ATRA-treated cells were responsive to depolarization and Na^+^ ion channel activation, suggesting that ATRA-induced neuron-like cells have excitable property similar to neurons isolated from canine cerebral cortex. In addition, the ATRA-treated cells voltage-dependently released neurotransmitter GABA and their gene expression profiles are similar to those of GABAergic cortical interneuron. Therefore, our simple and rapid method for the generation of GABAergic cortical interneuron-like cells may be useful not only as models of neuronal diseases that affect inhibitory neurons, but also for cell-type-specific drug screening.

Previous studies have reported chemical induction of neuronal cells, but the underlying mechanism for cell-type-specific reprogramming is still unclear [12–15]. Therefore, elucidating the underlying mechanism will provide new insights into the *in vitro* generation of autologous neurons. NGFR is expressed and plays a functional role in GABAergic neuron developed in the basal forebrain [49]. Furthermore, it has been reported that NGFR-deficient mice show significantly reduced telencephalic neurogenesis [50, 51]. In this study, the expression of NGFR was dramatically increased in DFATs treated with ATRA. Therefore, we used NGFR as a marker for ATRA-induced neuronal reprogramming and screened the inhibitors of several pathways for intracellular signaling. Our unbiased screening using RNA-seq-based pathway analysis showed that ATRA induced the activation of MAPK pathway, which was confirmed by chemical screening and knockdown experiment for NGFR mRNA expression. Consequently, we showed that JNK3, which is an uncharacterized JNK subtype abundant in the brain [52–54], played an important role in the initial stage of ATRA-induced neuronal reprogramming. Further studies examining JNK3 signaling may be important to elucidate the mechanisms through which ATRA stimulates the neuronal reprogramming of DFATs.

## Conclusions

In conclusion, our findings provide a new insight into the role of ATRA in neuronal reprogramming, which may contribute to the development of more efficient and precise neuronal reprogramming. The neuron-like cells from canine DFATs could also be a powerful tool for translational research in cell transplantation therapy, *in vitro* disease modeling, and screening of drugs for neuronal diseases.

## Supporting information

S1 Fig Characterization of cell surface marker of canine DFATs. Analysis of cell surface marker expression in DFATs by flow cytometry. DFAT cells were positive for mesenchymal lineage markers CD29, CD44 and CD90, but negative for the hematopoietic lineage markers CD14, CD45, HLA-DR and CD34. Solid and open histograms show non-specific and specific staining for the indicated marker, respectively.
(PDF)

S2 Fig The effect of ATRA on the neural stem cells or glial cells marker expression. (A) mRNA expression of the neural stem cell marker (NES) in DFATs treated with ATRA. Primary cultured neurons were used as a negative control (NC). (B) mRNA expression of the glial cell marker (GFAP) in DFATs treated with ATRA. Primary cultured glial cells were used as a positive control (PC). (C) Protein expression of the neuronal stem cell marker (NES; upper row) and glial cell marker (GFAP; middle row) in DFATs treated with ATRA. β-actin (lower row) was used as an internal standard.
(PDF)

S3 Fig ATRA induced the intrinsic neuronal reprogramming. (A) Gene ontology (GO) analysis of the main enriched genes after ATRA treatment. (B) Validation of the expression of neuronal cell markers by Real-time RT-PCR. (C) Heatmap showing differentially expressed genes (P < 0.05). The number above the heat map indicates independent biological replicates. The GO for each block is shown (as labeled on the left). Red and blue indicate upregulated and downregulated genes, respectively.
(PDF)

S4 Fig Gene ontology (GO) analysis of the four groups. The upregulated genes under GO terms of nervous system development were classified into four groups by unsupervised hierarchical cluster analysis. The typical neuronal marker genes (e.g. NEFH and NEFL) and related GO terms (e.g. neuron part, axon guidance, neurofilament and neurofilament cytoskeleton organization) were classified into group 1.
(PDF)

S5 Fig Uncropped images for the blots shown in Fig 1. (PDF)

S6 Fig Uncropped images for the blots shown in Fig 8. (PDF)
