# Supplementary material for: All-trans retinoic acid induces reprogramming of canine dedifferentiated cells into neuron-like cells
Source: PLoS One. 2020 Mar 31;15(3):e0229892. doi: 10.1371/journal.pone.0229892 (PMC7108708; doi:10.1371/journal.pone.0229892)
Supplement: S1 Fig — Analysis of cell surface marker expression in DFATs by flow cytometry. DFAT cells were positive for mesenchymal lineage markers CD29, CD44 and CD90, but negative for the hematopoietic lineage markers CD14, CD45, HLA-DR and CD34. Solid and open histograms show non-specific and specific staining for the indicated marker, respectively. (PDF) [file pone.0229892.s001.pdf]

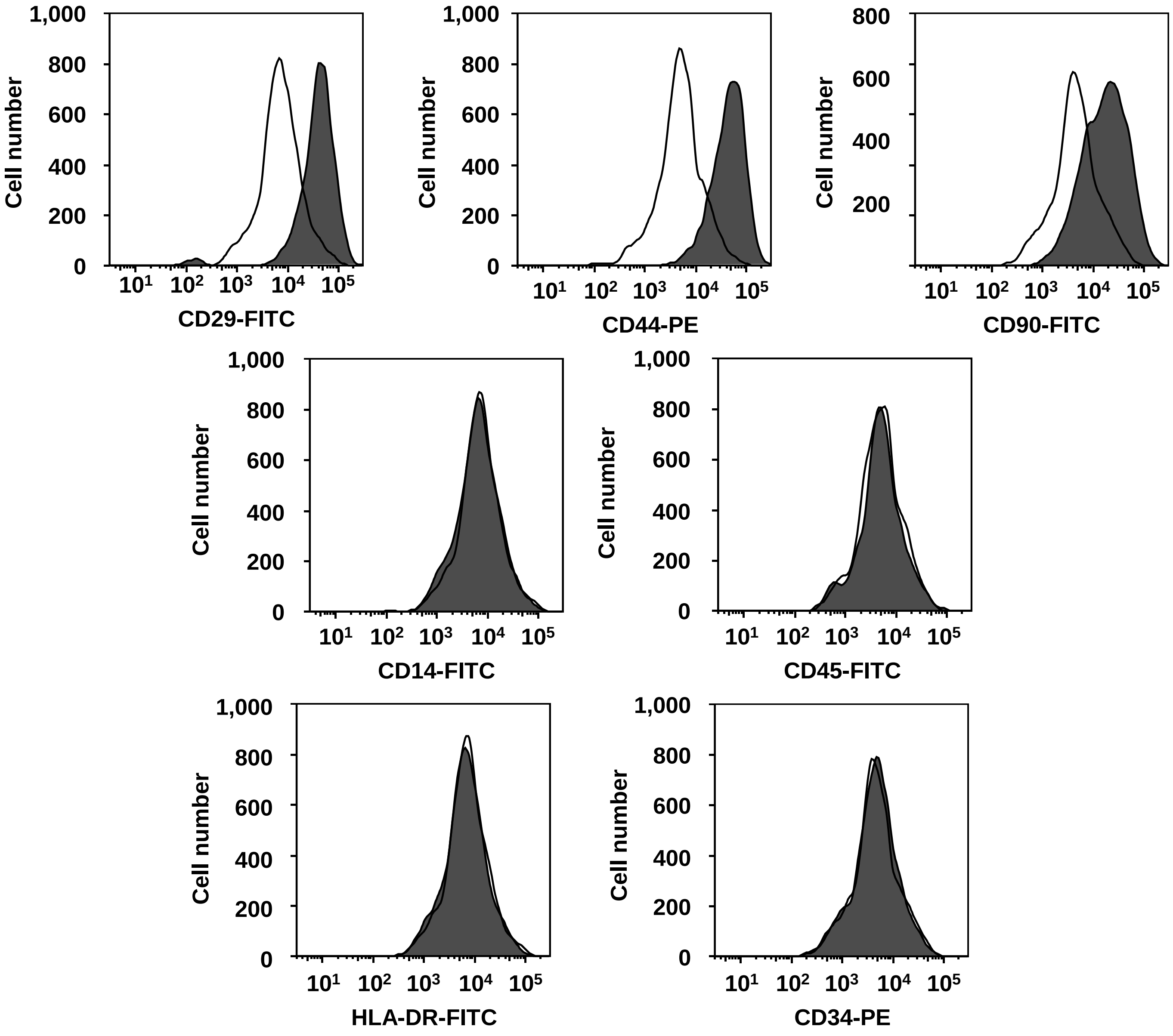

S1 Fig. Characterization of cell surface marker of canine DFATs.
Analysis of cell surface marker expression in DFATs by flow cytometry. DFAT cells were positive for mesenchymal lineage markers CD29, CD44 and CD90, but negative for the hematopoietic lineage markers CD14, CD45, HLA-DR and CD34. Solid and open histograms show non-specific and specific staining for the indicated marker, respectively.
