# Supplementary material for: All-trans retinoic acid induces reprogramming of canine dedifferentiated cells into neuron-like cells
Source: PLoS One. 2020 Mar 31;15(3):e0229892. doi: 10.1371/journal.pone.0229892 (PMC7108708; doi:10.1371/journal.pone.0229892)
Supplement: S2 Fig — (A) mRNA expression of the neural stem cell marker (NES) in DFATs treated with ATRA. Primary cultured neurons were used as a negative control (NC). (B) mRNA expression of the glial cell marker (GFAP) in DFATs treated with ATRA. Primary cultured glial cells were used as a positive control (PC). (C) Protein expression of the neuronal stem cell marker (NES; upper row) and glial cell marker (GFAP; middle row) in DFATs treated with ATRA. β-actin (lower row) was used as an internal standard. (PDF) [file pone.0229892.s002.pdf]

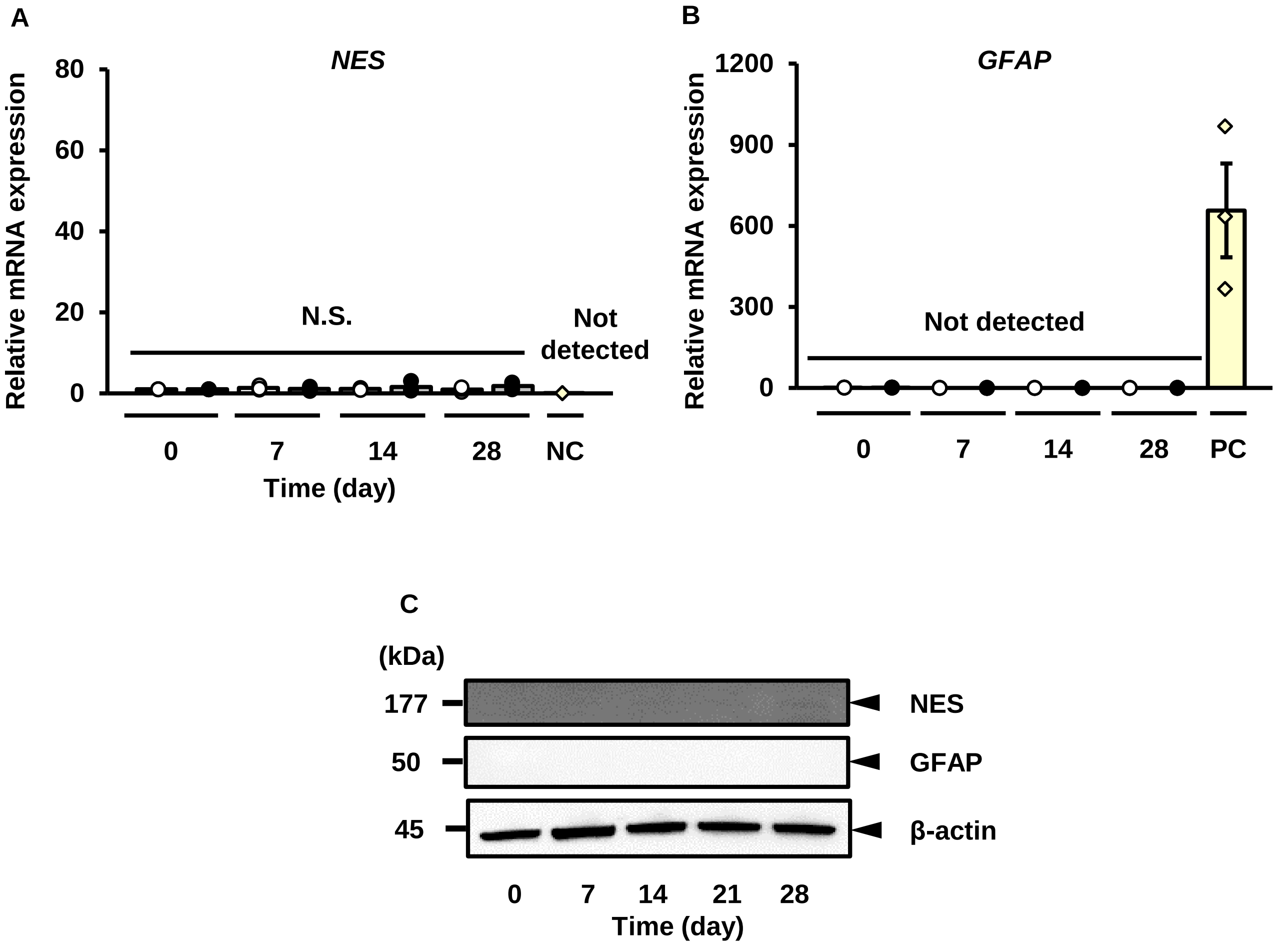

S2 Fig. The effect of ATRA on the neural stem cells or glial cells marker expression.

(A) mRNA expression of the neural stem cell marker (NES) in DFATs treated with ATRA. Primary cultured neurons were used as a negative control (NC).

(B) mRNA expression of the glial cell marker (GFAP) in DFATs treated with ATRA. Primary cultured glial cells were used as a positive control (PC).

(C) Protein expression of the neuronal stem cell marker (NES; upper row) and glial cell marker (GFAP; middle row) in DFATs treated with ATRA. β-actin (lower row) was used as an internal standard.
