# Supplementary material for: All-trans retinoic acid induces reprogramming of canine dedifferentiated cells into neuron-like cells
Source: PLoS One. 2020 Mar 31;15(3):e0229892. doi: 10.1371/journal.pone.0229892 (PMC7108708; doi:10.1371/journal.pone.0229892)
Supplement: S3 Fig — (A) Gene ontology (GO) analysis of the main enriched genes after ATRA treatment. (B) Validation of the expression of neuronal cell markers by Real-time RT-PCR. (C) Heatmap showing differentially expressed genes (P < 0.05). The number above the heat map indicates independent biological replicates. The GO for each block is shown (as labeled on the left). Red and blue indicate upregulated and downregulated genes, respectively. (PDF) [file pone.0229892.s003.pdf]

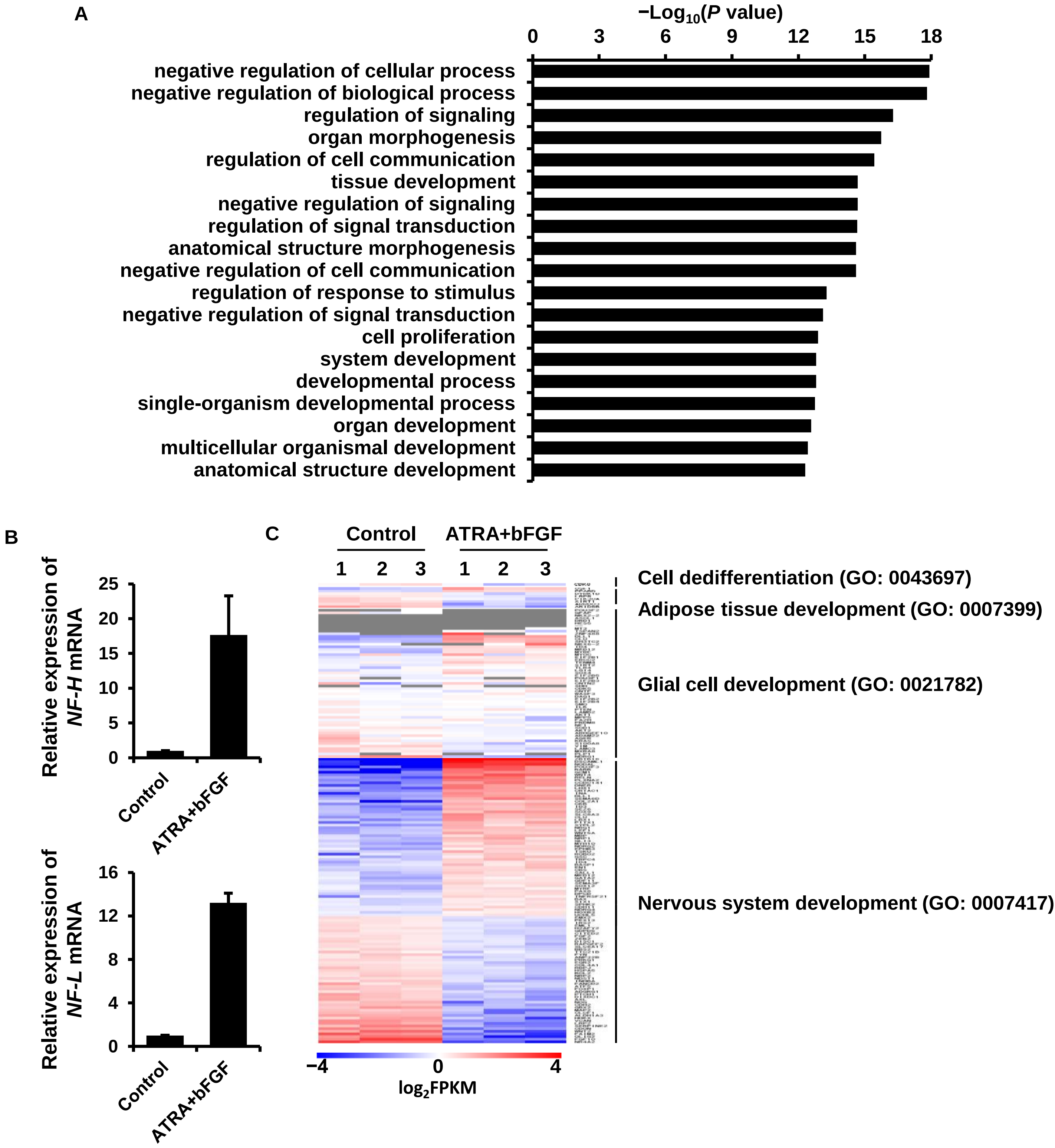

S3 Fig. ATRA induced the intrinsic neuronal reprogramming
(A) Gene ontology (GO) analysis of the main enriched genes after ATRA treatment.
(B) Validation of the expression of neuronal cell markers by Real-time RT-PCR.
(C) Heatmap showing differentially expressed genes ($P < 0.05$). The number above the heat map indicates independent biological replicates. The GO for each block is shown (as labeled on the left). Red and blue indicate upregulated and downregulated genes, respectively.
