# Supplementary material for: All-trans retinoic acid induces reprogramming of canine dedifferentiated cells into neuron-like cells
Source: PLoS One. 2020 Mar 31;15(3):e0229892. doi: 10.1371/journal.pone.0229892 (PMC7108708; doi:10.1371/journal.pone.0229892)
Supplement: S4 Fig — The upregulated genes under GO terms of nervous system development were classified into four groups by unsupervised hierarchical cluster analysis. The typical neuronal marker genes (e.g. NEFH and NEFL) and related GO terms (e.g. neuron part, axon guidance, neurofilament and neurofilament cytoskeleton organization) were classified into group 1. (PDF) [file pone.0229892.s004.pdf]

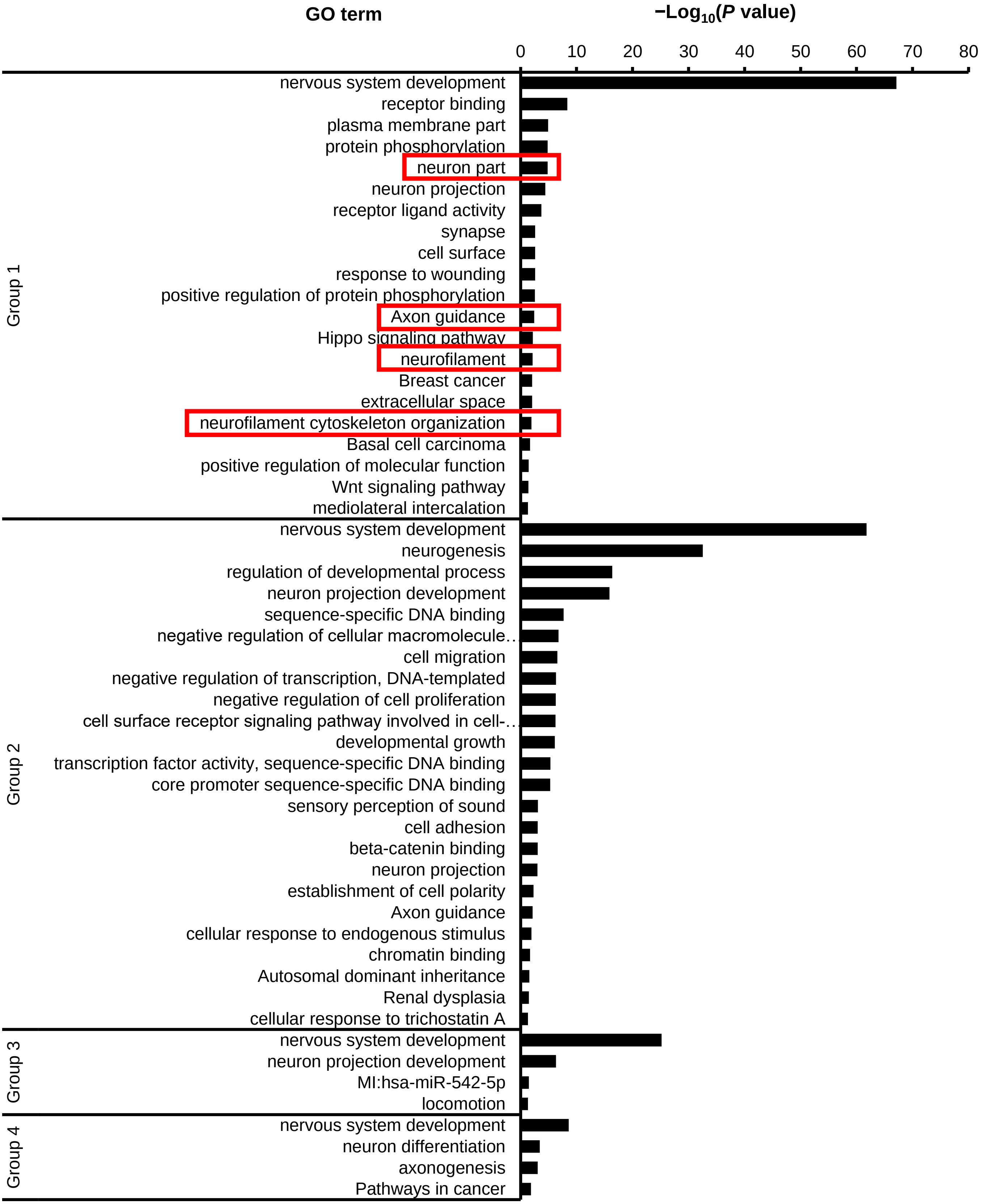

S4 Fig. Gene ontology (GO) analysis of the four groups. The upregulated genes under GO terms of nervous system development were classified into four groups by unsupervised hierarchical cluster analysis. The typical neuronal marker genes (e.g. NEFH and NEFL) and related GO terms (*e.g.* neuron part, axon guidance, neurofilament and neurofilament cytoskeleton organization) were classified into group 1.
