# Supplementary figures and images for: All-trans retinoic acid induces reprogramming of canine dedifferentiated cells into neuron-like cells
Source: PLoS One. 2020 Mar 31;15(3):e0229892. doi: 10.1371/journal.pone.0229892 (PMC7108708; doi:10.1371/journal.pone.0229892)

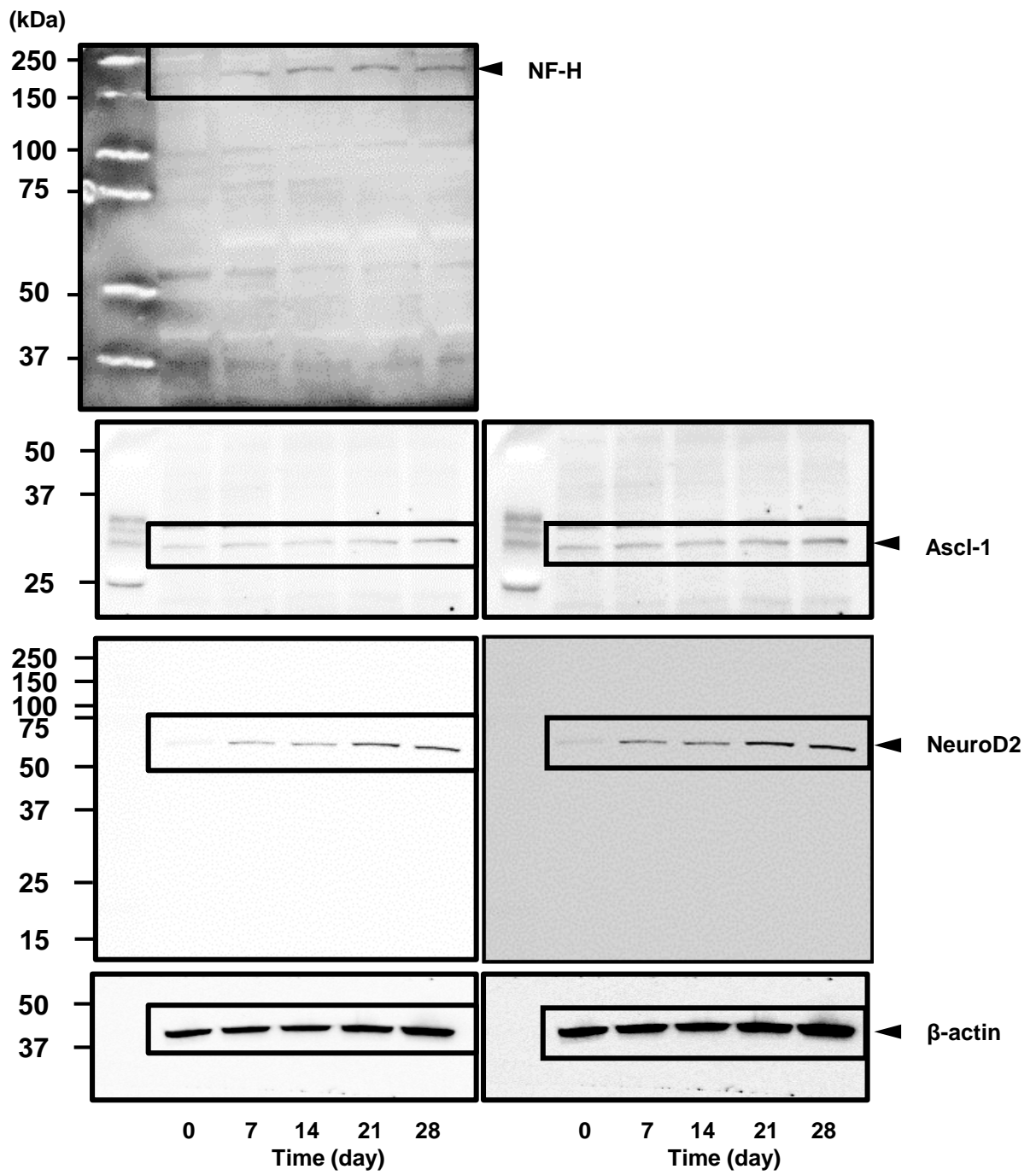

S5 Fig. Uncropped images for the blots shown in Fig. 1.

Supplement: S5 Fig — (PDF) [file pone.0229892.s005.pdf]

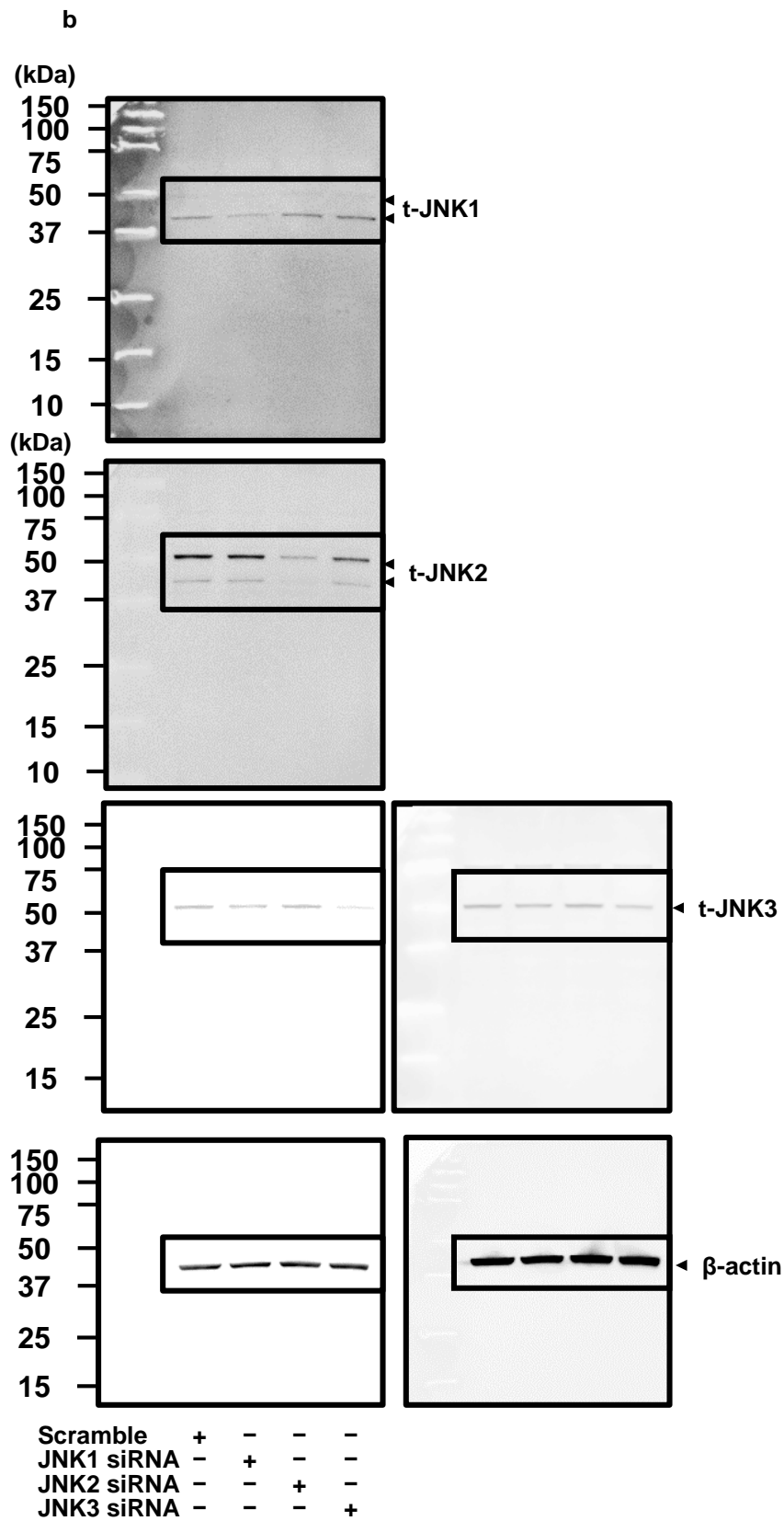

S6 Fig. Uncropped images for the blots shown in Fig. 8.

Supplement: S6 Fig — (PDF) [file pone.0229892.s006.pdf]
